# Green synthesis of C5–C6-unsubstituted 1,4-DHP scaffolds using an efficient Ni–chitosan nanocatalyst under ultrasonic conditions

**DOI:** 10.3762/bjoc.18.14

**Published:** 2022-01-25

**Authors:** Soumyadip Basu, Sauvik Chatterjee, Suman Ray, Suvendu Maity, Prasanta Ghosh, Asim Bhaumik, Chhanda Mukhopadhyay

**Affiliations:** 1 Department of Chemistry, University of Calcutta, 92 APC Road, Kolkata-700009, India; 2 Department of Materials Science, Indian Association for the Cultivation of Science, Jadavpur, Kolkata-700032, India; 3 Department of Chemistry, Presidency University, Kolkata-700073, India; 4 Department of Chemistry, R. K. Mission Residential College, Narendrapur, Kolkata-700103, India

**Keywords:** 1,4-DHPs, green synthesis, magnetically recyclable catalyst, Ni–chitosan nanoparticles, ultrasonication

## Abstract

A heterogeneous and magnetically recyclable Ni–chitosan nanocatalyst was synthesized and thoroughly characterized by powder Fourier-transform infrared (FTIR) spectroscopy, X-ray diffraction (XRD) analysis, scanning electron microscopy (SEM), high-resolution transmission electron microscopy (HRTEM), energy-dispersive X-ray (EDX) spectroscopy, etc. It was effectively utilized in the eco-friendly synthesis of new C5–C6-unsubstituted 1,4-DHPs under ultrasonic irradiation. The important focus of the methodology was to develop an environmentally friendly protocol with a short reaction time and a simple reaction procedure. The other advantages of this protocol are a wide substrate scope, a very good product yield, the use of an eco-friendly solvent and a recyclable nanocatalyst, as well as reaction at room temperature.

## Introduction

Homogeneous catalysts, despite having an outstanding application in the field of synthetic chemistry, suffer various notable disadvantages, such as difficulties in the recovery and reuse of the catalyst. These drawbacks can cause both economic and environmental concerns that strongly reduce the applicability in various organic syntheses. Therefore, efforts towards heterogenization of the catalytic systems used in the field of environmental chemistry keep growing day by day. These allow complete separation of novel or harmful catalysts from the mixture and reuse for further treatment. This makes the whole catalytic processes more efficient and cost-effective for industrial purposes [1–3]. In this regard, different insoluble supports, such as alumina [4], silica [5], zeolites [6], polymers [7], carbon nanotubes [8], etc. have been reported. Recently, the use of bio-based natural polymers became one of the interesting features of heterogeneous catalysts. Chitosan is one of the most abundant bio-based polymers in nature [9]. It is the N-deacetylated form of chitin found in industrial waste [10]. Chitosan is a broadly used natural polymer because of properties such as biocompatibility, low cost, and nontoxicity. It has diverse applications, such as drug delivery [11], biomedical uses [12], removal of toxic metals from wastewater [13], in manufacturing processes [14], in the food industry [15], in agriculture [16], and as catalyst in transesterification reactions [17]. Chitosan is regarded as one of the most effective bio-based polymers to chelate transition metal ions due to the presence of abundant amino and alcohol groups in the structure [18]. Because of this chelating character, as well as due to the hydrophilicity, unique three-dimensional structure, and mechanical properties, chitosan has several catalytic applications [19–20]. In this work, we synthesized a Ni–chitosan complex and exploited the coordination properties of the complex to use it as an effective and recyclable catalyst towards the green synthesis of C5–C6-unsubstituted 1,4-dihydropyridine (1,4-DHP) scaffolds.

Ultrasonic irradiation is an important technique in the toolbox of green chemistry [21]. The application of ultrasound in “traditional” reactions results in a lower reaction time, higher conversion, and simpler methodology and is termed sonochemistry. Ultrasonication is a modern trend in synthetic chemistry that supports the objective of green chemistry, namely the reduction of the environmental effects of chemical synthesis [22]. The use of ultrasonic irradiation allows for optimal mixing of the reactants and the catalyst by enhancing the homogeneity of the reaction medium. It also decreases the chance of agglomeration of the heterogeneous nanoparticles (NPs) and therefore automatically increases the dispersibility of the catalyst throughout the medium, resulting in a higher catalytic activity [23].

1,4-DHPs are considered one of the most useful molecular scaffolds in medicinal chemistry. The scaffold is the main constituent of several crucial drugs, including amlodipine and nifedipine [24]. The structure of 1,4-DHP resembles the coenzyme NADH, which is very important in oxidation and reduction reactions in biological systems. 4-Substituted 1,4-DHPs have been used in the treatment of cardiovascular diseases as organic calcium channel modulators [25], which further increases the importance of this compound class. Much work has been dedicated to the synthesis of 1,4-DHPs, but in most of these reports, the DHP ring is fully substituted. There are only few methods available for the synthesis of C5–C6-unsubstituted 1,4-DHPs. The presence of an unsubstituted double bond next to the nitrogen atom permits a DHP to react as an enamine, for further transformation to complex heterocyclic scaffolds [26]. The exposed double bond can also undergo modification to produce different kinds of moieties. There are only few reports on the synthesis of C5–C6-unsubstituted 1,4-DHPs, to the best of our knowledge. However, no previous report on the synthesis of the above-mentioned moiety was found using a magnetically separable Ni–chitosan nanocatalyst. Further, the use of ultrasonic irradiation, allowing for a very short reaction time and a very good yield, undoubtedly highlights the importance of this work.

In this work, we attempted to highlight the synthesis of 5,6-unsubstituted 1,4-DHPs, which are quite rarely found in the literature in comparison to simple 1,4-DHPs, which are quite common. There are many examples for the synthesis of conventional 1,4-DHPs in the literature, which generally involve four components. These include one primary amine, two multiple bonds, and one aldehyde function [27]. However, one advantage of our study is that we only used three components since the cinnamaldehyde derivatives **3** played the role of two components at the same time, namely that of an aldehyde and that of one multiple bond. Generally, aldehydes and multiple bonds are very reactive in the presence of primary amines. However, since we used cinnamaldehyde derivatives **3**, which are conjugated systems of a double bond and an aldehyde, the reactivity was rather low compared to other aldehydes. Thus, herein we had to use a metal catalyst to increase the reactivity of the carbonyl moiety. Moreover, the catalyst was thoroughly characterized by various methods and is easily recyclable.

## Results and Discussion

### Characterization of the catalyst

**Figure 1:**
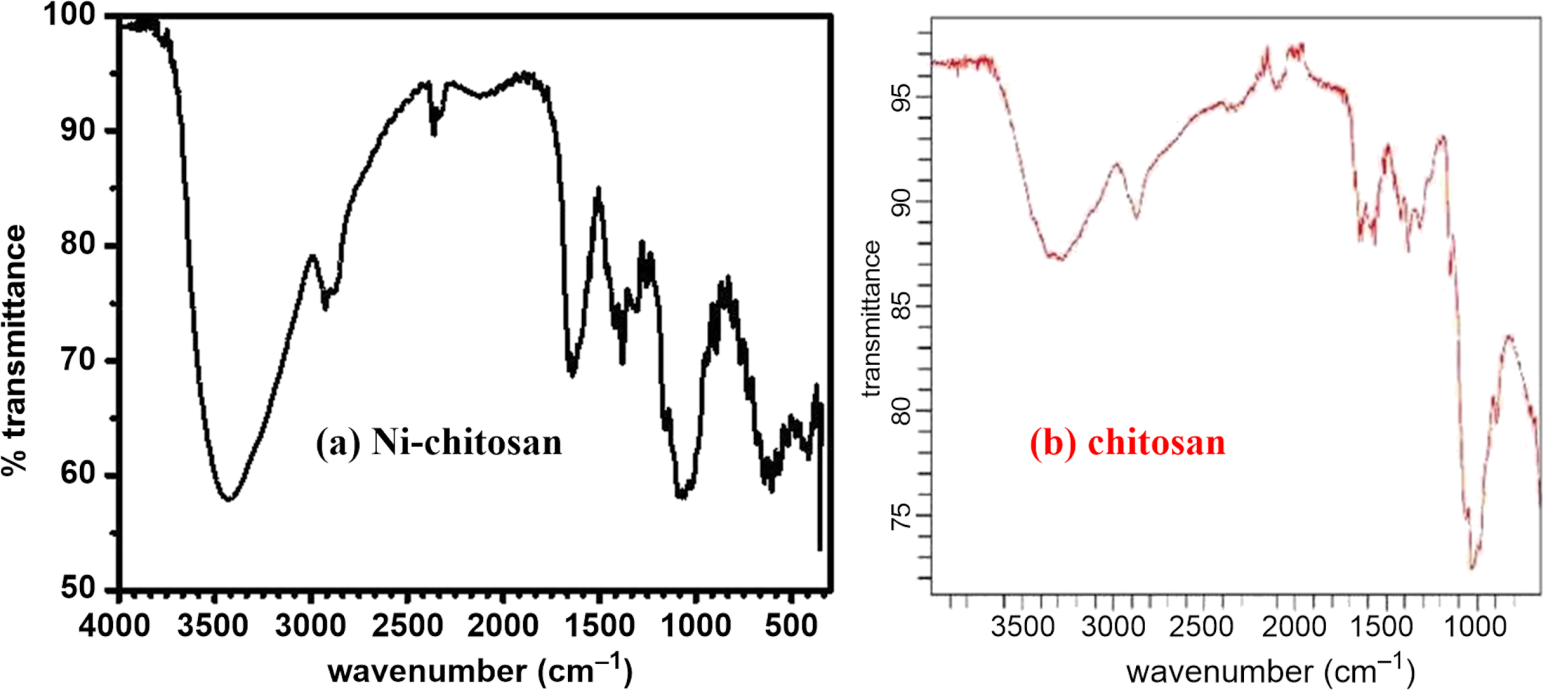
FTIR spectra of (a) the Ni–chitosan NPs and (b) bare chitosan.

An FTIR analysis of bare chitosan was carried out, and the spectrum exhibited characteristic peaks for the O–H and N–H stretching vibrations in the range of 3300–3400 cm^−1^ (Figure 1b). For the chitosan-supported nickel catalyst, the band around 3400 cm^−1^ became much sharper and stronger compared to bare chitosan (Figure 1a). The spectroscopic FTIR study revealed the interaction between the metal and the NH_2_ and OH groups of chitosan. In both spectra, the peaks around 1100, 1400, 1600, and 2900 cm^−1^ corresponded to the C–O, C–N, and N–H (bending) as well as to the C–H bonds of the chitosan moiety, respectively. The similarity of the two spectra may have been due to the low content of nickel in the catalyst.

**Figure 2:**
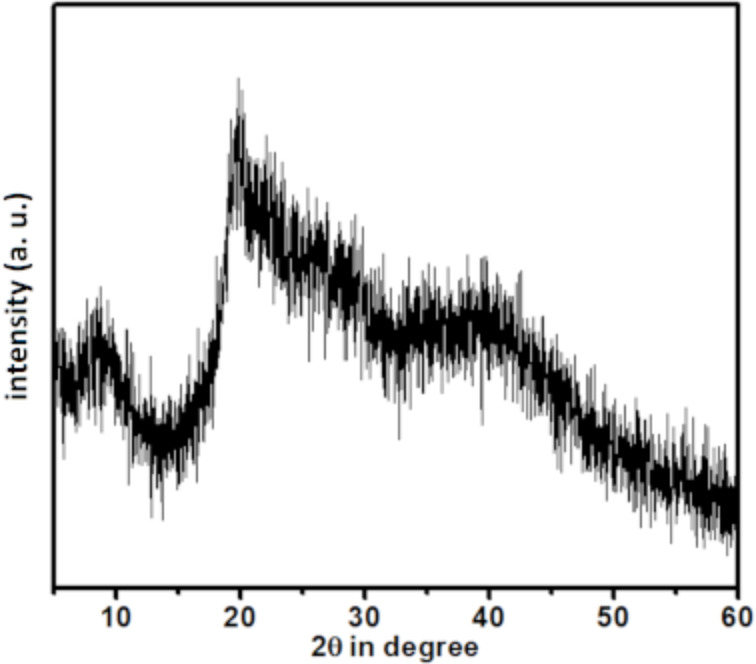
PXRD data for the Ni–chitosan NPs.

To understand the crystallinity of the material, we carried out a powder XRD (PXRD) analysis (Figure 2). Chitosan, in general, gives rise to a characteristic, partially crystalline phase by virtue of intramolecular H-bonding. The presence of diffraction peaks at 2Θ 9.2 and 19.7 (in degree) indicated the presence of a chitosan framework [28], whereas the other minor diffraction peaks were observed due to the presence of Ni(II).

**Figure 3:**
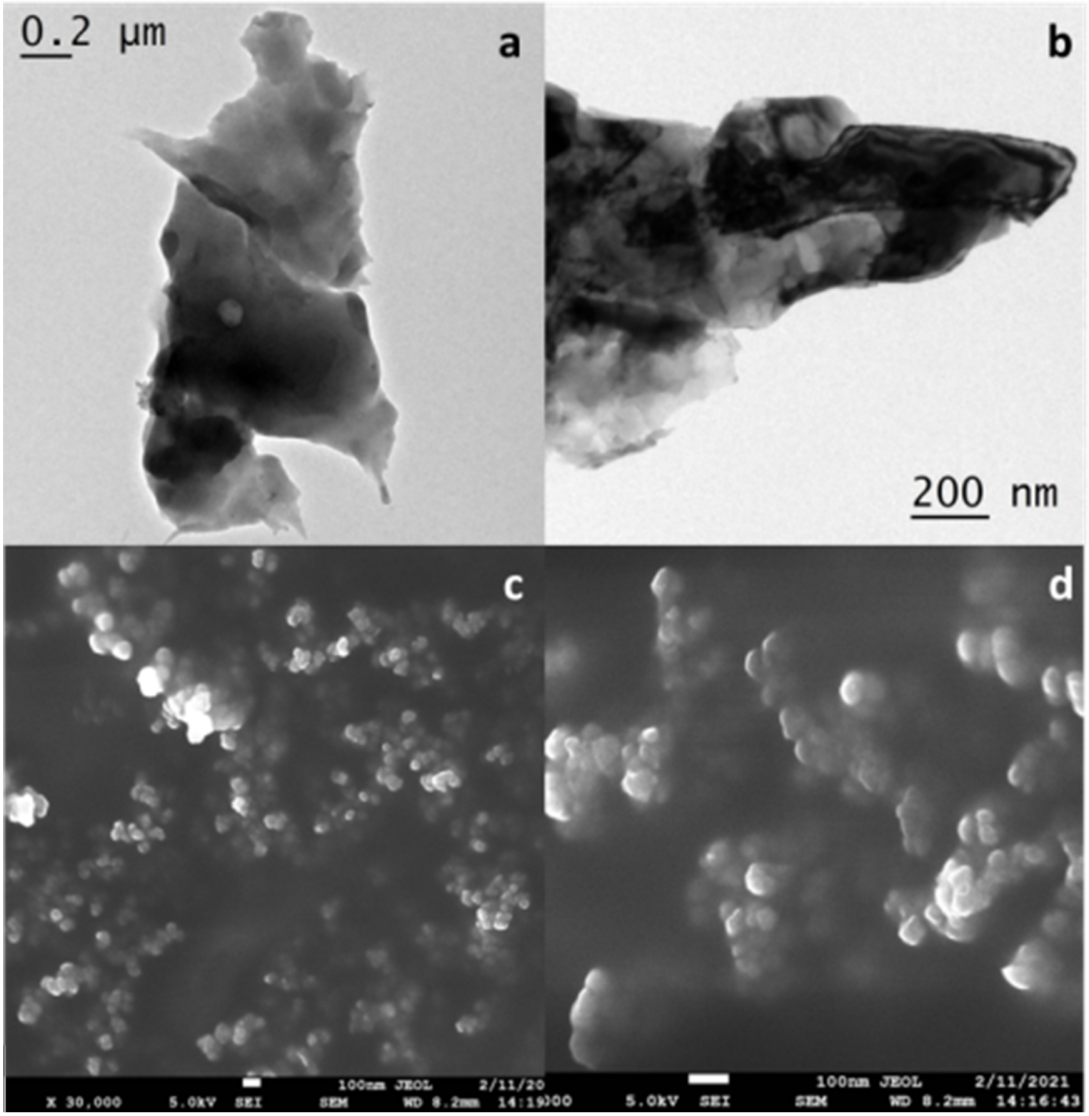
TEM (a and b) and SEM images (c and d) of the Ni–chitosan NPs.

From the SEM and TEM images of the catalyst, we confirmed the morphological features of the material (Figure 3a and Figure 3b). The TEM images showed agglomeration of spherical NPs, leading to the formation of multiple scaffolds. In the SEM images, the presence of spheres of 40–60 nm was clearly visible (Figure 3c and Figure 3d). Often, these small NPs were agglomerated to form bigger particles of over 100 nm. The agglomeration of the TEM samples occurred upon solvent evaporation after the sample had been drop-casted on the carbon-coated Cu grid. This might have happened due to specific surface interactions of the particles. On the other hand, the SEM sample was prepared under dry conditions. As such, we observed different particle sizes in the SEM and TEM analyses. Since small particles of 40–60 nm were present consistently throughout the grid, they fell within the definition of NPs.

**Figure 4:**
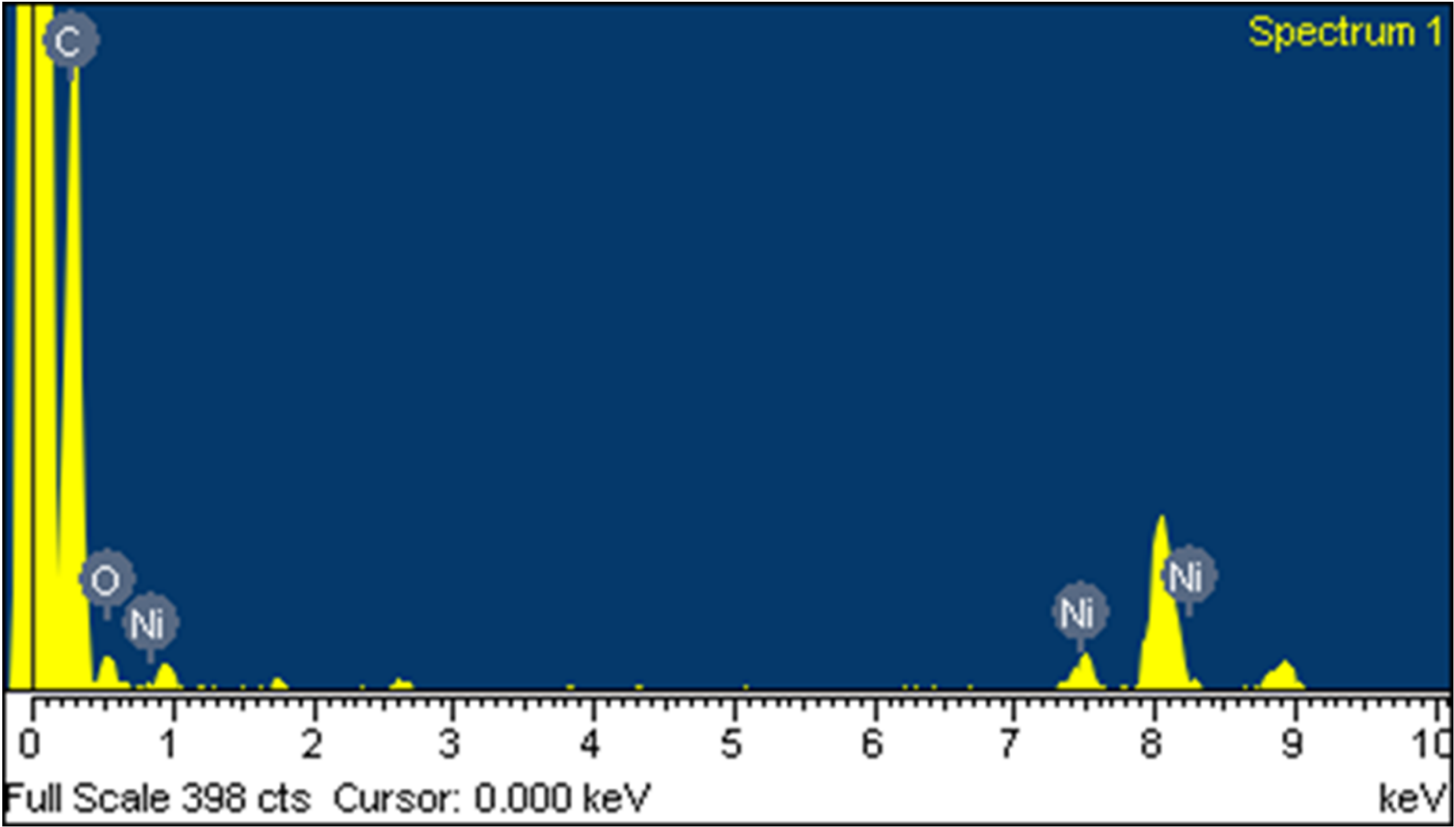
EDX spectrum of the Ni–chitosan NPs.

The EDX study of the catalyst (Figure 4) confirmed the presence of nickel in this sample. In the spectrum, the peaks for carbon and nitrogen appeared at very close energy values. Since the amount of nitrogen was small, the corresponding peak may have been overlaid by that of carbon, so that both could not be distinguished clearly. To avoid an inaccurate estimation of the elemental composition, nitrogen was therefore not marked separately. Rather, the total contribution of carbon and nitrogen was summarized.

We performed an inductively coupled plasma–optical emission spectroscopy (ICP–OES) analysis of the catalyst for the determination of the nickel loading. The obtained data is given in Table 1.

**Table 1:** ICP–OES analysis of the Ni–chitosan NPs.

element, wavelength (nm)	weight of sample/volume	dilution factor	concentration in ppm

Ni, 231.604	0.0071 g/50 mL	1	3.215 mg/L


[1] $$\begin{array}{c} \text{wt \% (g/}100\text{ g) = }\frac{\text{instrument value (ppm)}\cdot \text{volume (m​L)}\cdot \text{dilution factor}\cdot {\text{10}}^{-\text{4}}}{\text{wt of sample (g)}}\\ =\frac{3.215\cdot 50\cdot 1\cdot 10^{-4}}{0.0071}\\ =2.264\% \end{array}$$

Thus, according to Equation 1, the nickel loading of the catalyst was found to be 2.264 wt % by ICP–OES analysis.

### Standardization of reaction conditions

Due to the remarkable medicinal activity of 1,4-DHPs, we became interested in developing a simple synthetic route to this type of moiety. We mainly focused on the synthesis under green aspects and towards a high product diversity. We initially chose three green solvents with a relatively low boiling point, ethanol, water, and acetone, respectively, as a reaction medium. A series of reactions was examined in these solvents, using an equimolar mixture of *p*-toluidine, *trans*-cinnamaldehyde, and dimethyl but-2-ynedioate, applying both stirring at room temperature and ultrasonication, respectively, in the presence of the synthesized catalyst. For all solvents, the result was much better under sonication compared to stirring at room temperature. In the ultrasonication procedure, the reaction was started at room temperature, and after completion of the reaction, the final temperature of the solution was found to be 40 °C. Among the solvents, ethanol afforded the highest yield under sonication within a very short reaction time. When we varied the reaction time, it was found that the highest yield was achieved with 15 minutes of ultrasonic irradiation (Table 2, entry 10). The use of chitosan (Table 2, entry 12) as a catalyst resulted only in traces of the product. We also used various Lewis acids, such as NiCl_2_, ZnCl_2_, and FeCl_3_ (Table 2, entries 13–15). A moderate to low yield was obtained when using these Lewis acids. To identify the optimal quantity of the catalyst, we altered the amount in several model reactions. It was found that the highest yield was obtained when 30 mg of the catalyst was used (Table 2, entry 17), and further increasing this quantity did not result in an enhanced yield. As such, the optimized conditions were sonication for 15 min in ethanol using 30 mg of the Ni–chitosan NPs (Table 2, entry 17). The optimization studies using this standard amount of catalyst are summarized in Table 2.

**Table 2:** Optimization of the reaction conditions.^a^

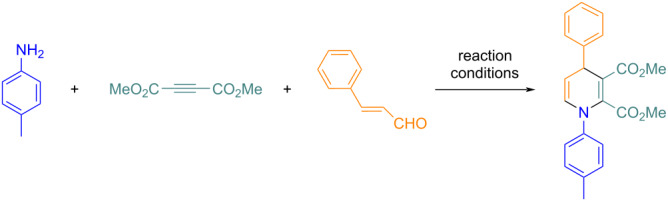						

entry	catalyst	amount of catalyst (mol % of Ni)	solvent	conditions	time	yield (%)^b^

1	Ni–chitosan NPs	40 mg (1.54 mol %)	EtOH	stirring (rt)	4 h	42
2	Ni–chitosan NPs	40 mg (1.54 mol %)	acetone	stirring (rt)	4 h	34
3	Ni–chitosan NPs	40 mg (1.54 mol %)	H_2_O	stirring (rt)	4 h	22
4	Ni–chitosan NPs	40 mg (1.54 mol %)	EtOH	ultrasound	10 min	68
5	Ni–chitosan NPs	40 mg (1.54 mol %)	acetone	ultrasound	10 min	40
6	Ni–chitosan NPs	40 mg (1.54 mol %)	H_2_O	ultrasound	10 min	52
7	Ni–chitosan NPs	40 mg (1.54 mol %)	EtOH	ultrasound	6 min	52
8	Ni–chitosan NPs	40 mg (1.54 mol %)	EtOH	ultrasound	8 min	58
9	Ni–chitosan NPs	40 mg (1.54 mol %)	EtOH	ultrasound	12 min	74
10	Ni–chitosan NPs	40 mg (1.54 mol %)	EtOH	ultrasound	15 min	88
11	Ni–chitosan NPs	40 mg (1.54 mol %)	EtOH	ultrasound	20 min	88
12	chitosan	40 mg	EtOH	ultrasound	20 min	trace
13	NiCl_2_	20 mol %	EtOH	ultrasound	15 min	42
14	ZnCl_2_	20 mol %	EtOH	ultrasound	20 min	28
15	FeCl_3_	20 mol %	EtOH	ultrasound	20 min	36
16	Ni–chitosan NPs	50 mg (1.93 mol %)	EtOH	ultrasound	15 min	88
17	Ni–chitosan NPs	30 mg (1.16 mol %)	EtOH	ultrasound	15 min	88
18	Ni–chitosan NPs	25 mg (0.96 mol %)	EtOH	ultrasound	15 min	70

^a^Reaction conditions: *p*-toluidine (1 mmol), dimethyl but-2-ynedioate (1 mmol), and *trans*-cinnamaldehyde (1 mmol). ^b^Isolated yield.

### Substrate scope

**Figure 5:**
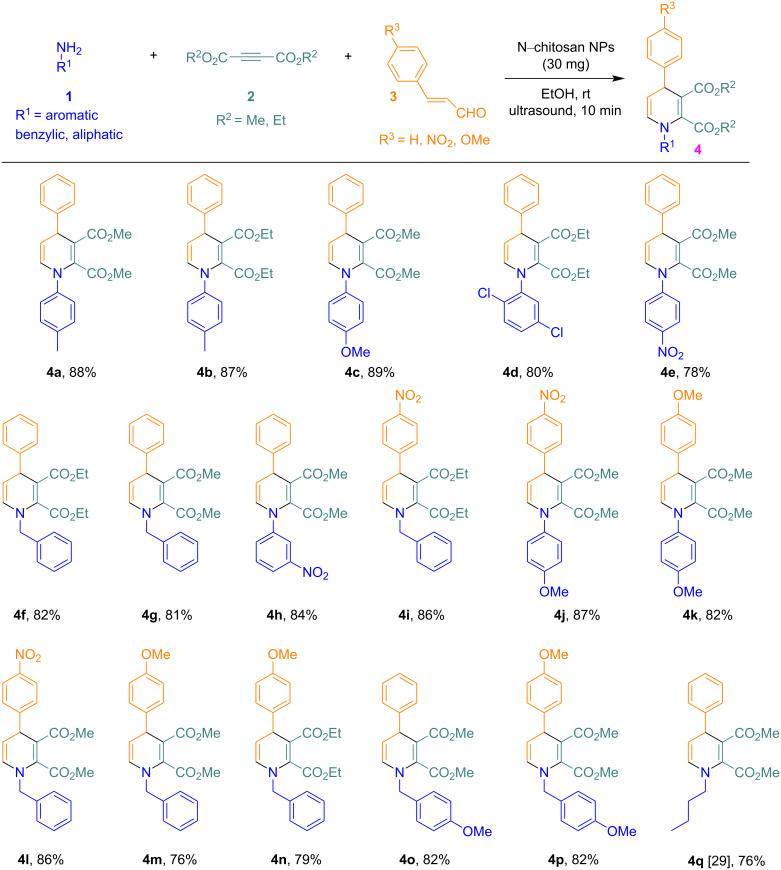
Synthesis of dialkyl 1,4-dihydropyridine-2,3-dicarboxylate derivatives.

The substrate scope and the generality of the reaction under the optimized conditions were explored through the synthesis of various products using differently substituted primary amines **1**, cinnamaldehydes **3**, and dialkyl but-2-ynedioates **2**. Aromatic, aliphatic, benzylic, and various other types of primary amines **1** afford an excellent yield. *Ortho*-, *meta*-, and *para*-substituted aromatic amines with both electron-donating and -withdrawing groups were used in this protocol. Aromatic amines with electron-donating groups offered a slightly higher yield compared to those with electron-withdrawing groups. *Para*-substituted cinnamaldehydes with both electron-donating and -withdrawing groups also resulted in a very good yield under the optimized conditions. Under the reaction conditions, both methyl- and ethyl-substituted but-2-ynedioates **2** were well tolerated. A total of 17 new compounds were synthesized with this methodology, as shown in Figure 5.

**Figure 6:**
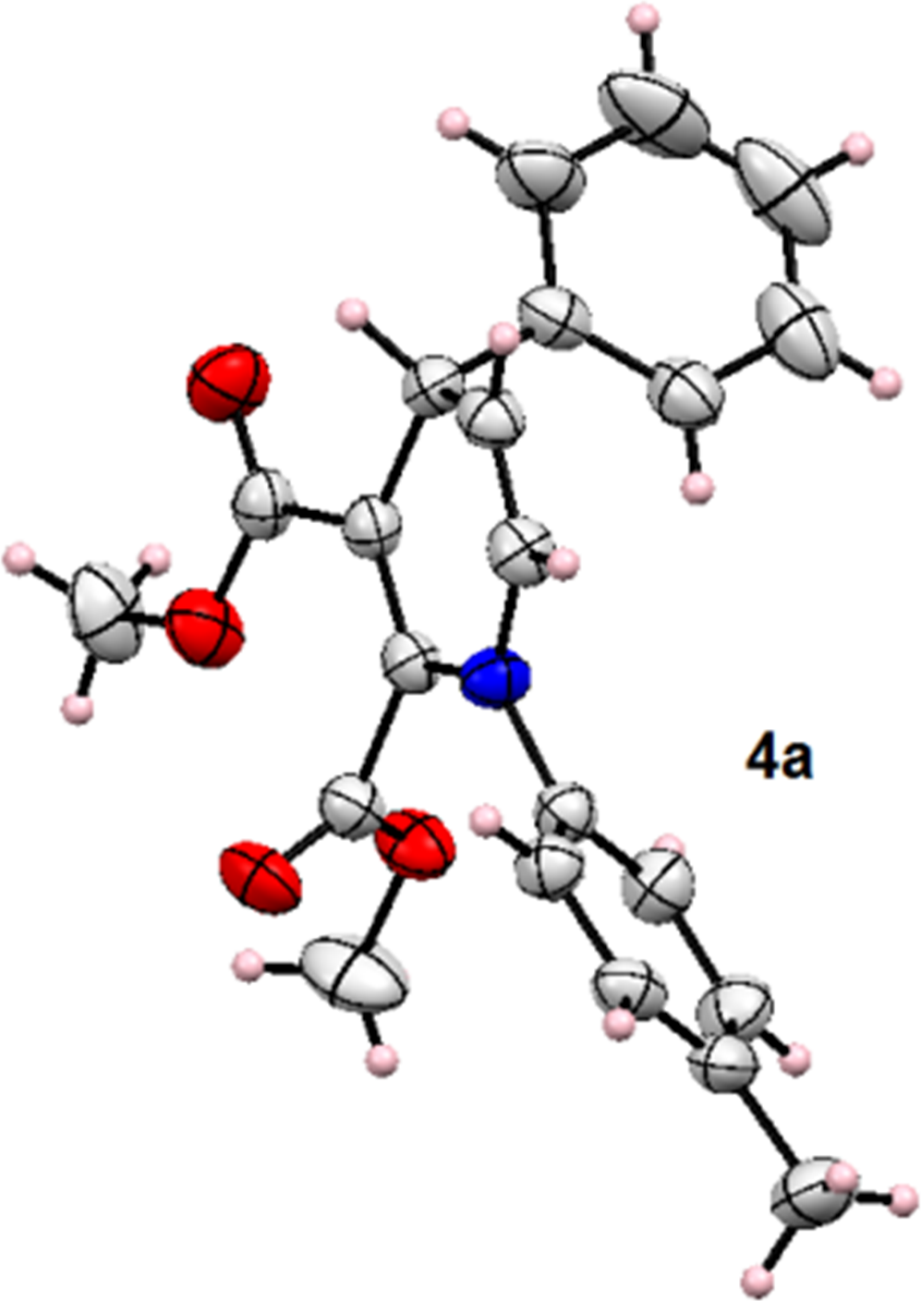
ORTEP representation of product **4a** (CCDC 1949329).

The synthesized products were characterized by ^1^H NMR, ^13^C NMR, HRMS, and melting point analysis. The structure of the compounds was also confirmed by single-crystal XRD analysis of **4a** (CCDC1949329, Figure 6).

### Plausible mechanism

**Scheme 1:**
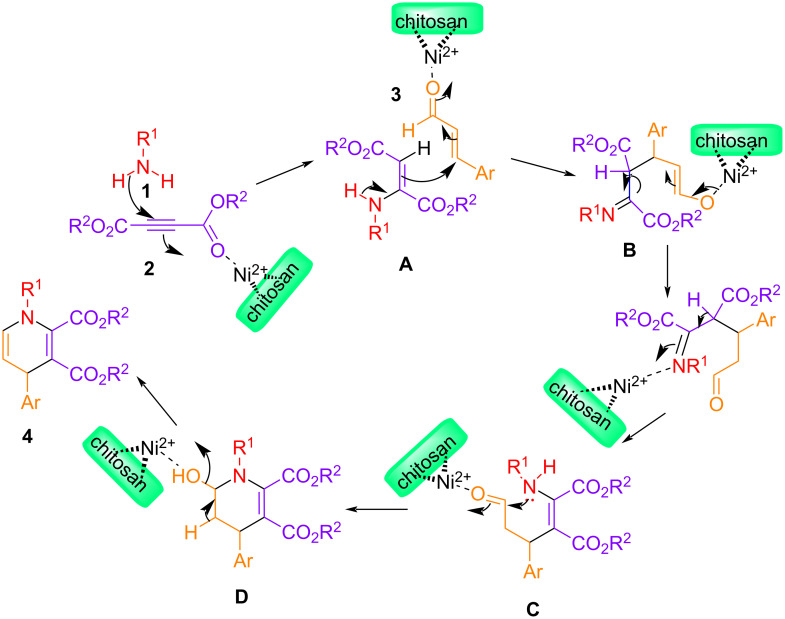
A plausible mechanistic route for the synthesis of C5–C6-unsubstituted 1,4-DHP derivatives using the Ni–chitosan nanocatalyst.

A plausible reaction mechanism for the synthesis of C5–C6-unsubstituted 1,4-DHPs is described in Scheme 1.

The reaction initiates with the formation of an enamine moiety (see **A**) by the reaction of a primary amine **1** and a but-2-ynedioate **2**. We isolated the intermediate **A** in the form of **4fA** [30], corresponding to compound **4f**, and the complete NMR data can be found in Supporting Information File 1. This enamine moiety reacts with a cinnamaldehyde compound **3** to give the desired product. Here, the role of the catalyst is to activate the cinnamaldehyde species **3**. The nickel of the catalyst coordinates to the oxygen atom of the cinnamaldehyde molecule **3**, enhancing the reactivity. The reaction between the enamine and the cinnamaldehyde derivative **3** advances via two steps: Initially, the enamine attacks the cinnamaldehyde compound **3** at the double bond and undergoes 1,4-addition to give intermediate **B**. The enol form is readily converted to the more stable aldehyde form **C**. In the second step, the nitrogen atom of the enamine function attacks the aldehyde carbon atom of the cinnamaldehyde unit in **3**, and one water molecule is eliminated to give the desired product. Both steps are accelerated by the presence of the catalyst. In support of our mechanistic pathway, we included a mass spectrum of the crude mixture of compounds **4k** and **4m** in Supporting Information File 1. Both spectra point at the presence of the respective intermediate **A** (in the form of **4kA** and **4mA**) and intermediate **D** (in the form of **4kD** and **4mD**).

### Recycling experiment of the Ni–chitosan nanocatalyst

**Figure 7:**
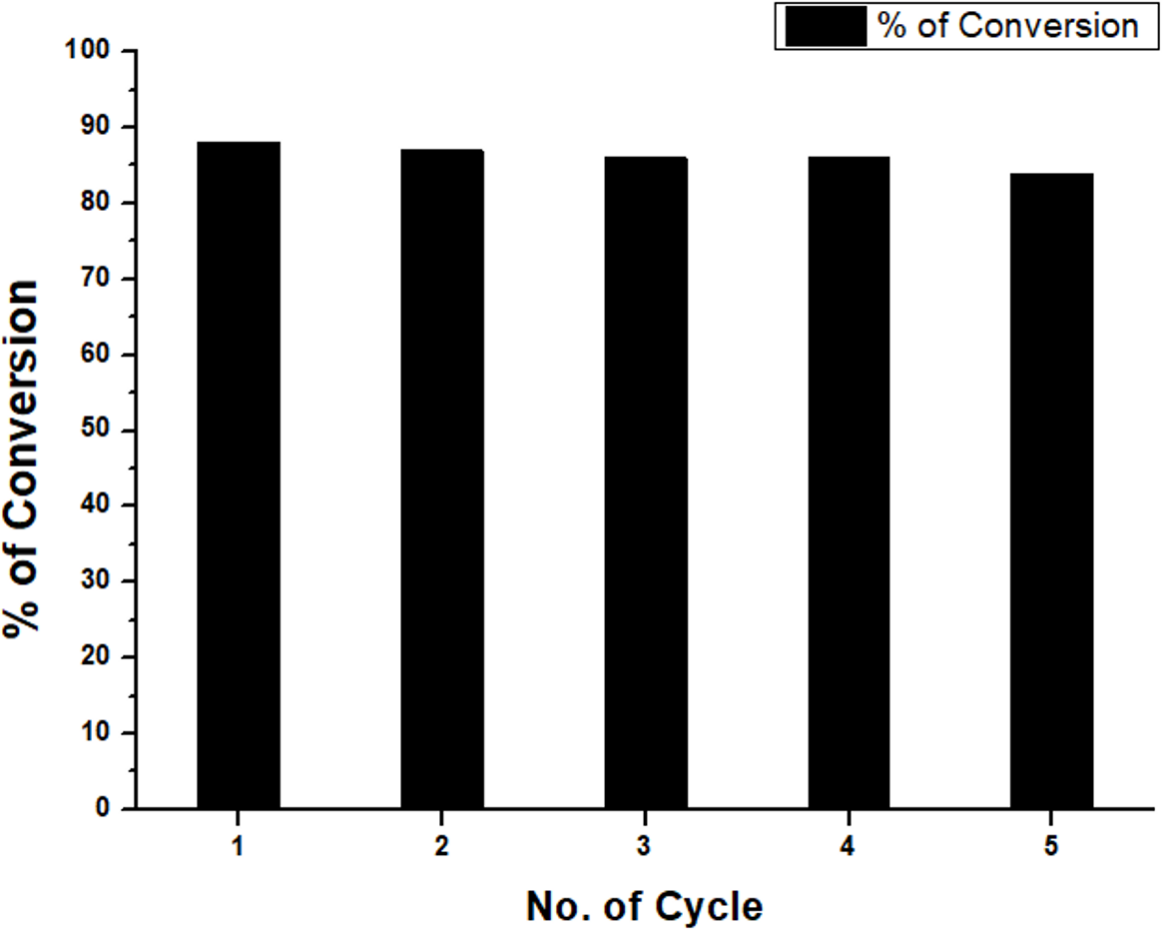
Recycling experiment of the Ni–chitosan nanocatalyst.

The reusability of the catalyst was studied to validate the catalytic adaptability of the Ni–chitosan nanocatalyst. A model reaction for the synthesis of **4a** was monitored, using the catalyst under the optimized conditions. After each run, the catalyst was recovered in up to 97% using an external magnet. In a series of five reactions, the catalyst was used repeatedly without significant decrease in catalytic activity (Figure 7). The slight reduction of the yield in later reactions may have been a result of the loss of catalyst in the recycling process.

### Comparison between present work and previously reported syntheses

A summarized comparison with previous reports on the synthesis of C5–C6-unsubstituted 1,4-DHPs is shown in Table 3. In all of the three previous studies, a longer reaction time or a higher temperature was required, or a lower product yield was noted. In contrast, our work unites various aspects of green chemistry, such as a minimal reaction time, a high conversion rate, a green solvent, environmentally friendly reaction conditions, and an effortless separation and recyclability of the catalyst. Altogether, the detailed summary in Table 3 highlights the advantages of employing the Ni–chitosan NPs for the synthesis of C5–C6-unsubstituted 1,4-DHPs under ultrasonication compared to the previous methodologies.

**Table 3:** Comparison between the previous reports on the synthesis of C5–C6-unsubstituted 1,4-DHPs and the present work.

entry	reaction conditions	solvent	time	temperature	catalyst	yield (%)	reference

1	heating	PhCN	36 h	50 °C	chiral phosphorus complex	82	[31]
2	heating (reflux)	H_2_O/DMF	10 h	85 °C	TMSCl	67	[32]
3	stirring	EtOH	6 h	rt	nanosized CuO	60	[33]
4	ultrasonication	EtOH	15 min	rt	Ni–chitosan NPs	88	present work

## Conclusion

In this protocol, our aim was to develop an environmentally friendly green methodology for the synthesis of biologically important 1,4-DHPs. Under these aspects, we utilized a heterogeneous and magnetically separable Ni–chitosan nanocatalyst under ultrasonic radiation in a green solvent to develop a new eco-compatible synthesis. A small amount of catalyst was required for the reaction, and the catalyst could be reused in up to five consecutive reactions without significant reduction of catalytic activity. To the best of our knowledge, no previous work is known using a Ni–chitosan nanocatalyst for the synthesis of C5–C6-unsubstituted 1,4-DHPs.

## Experimental

### Materials and instrumentation

All starting materials were purchased from commercial sources and used without further purification. Medium-molecular-weight chitosan (75–85% deacetylated) from Sigma-Aldrich, nickel(II) chloride hexahydrate (97% purity) from Loba Chemie, as well as *trans*-cinnamaldehyde (98+% purity), 4-nitrocinnamaldehyde (predominantly *trans*, 98% purity), and *trans*-4-methoxycinnamaldehyde (98+% purity) from Alfa Aesar were used. Dimethyl acetylenedicarboxylate (99% purity) and diethyl acetylenedicarboxylate (95% purity) were bought from Sigma-Aldrich. *p*-Toluidine (99% purity for synthesis), *p*-anisidine (99% purity for synthesis), 2,4-dichloroaniline (98% extra pure), *p*-nitroaniline (98.5% extra pure), *m*-nitroaniline (99% extra pure), and *n*-butylamine (98% purity) were bought from Loba Chemie. Benzylamine (99% purity) and *p*-methoxybenzylamine (98% purity) were bought from Sigma-Aldrich. The reactions were performed under sonication in a TAKASHI ultrasonic cleaning bath, and the progress of the reactions was monitored by TLC analysis using silica gel. ^1^H and ^13^C NMR spectra were recorded on Bruker 300 MHz and 400 MHz instruments using CDCl_3_, with TMS as internal reference. Melting points were recorded on an electrical melting point apparatus with an open capillary. XRD analysis was performed on a Bruker SMART diffractometer. PXRD data was recorded using a Bruker AXS D8 Advance SWAX diffractometer with Cu Kα (λ = 0.15406 nm) radiation. HRTEM data was obtained using a JEOL JEM 2010 transmission electron microscope. SEM data was obtained on a Hitachi S-5200 field-emission scanning electron microscope. FTIR data was recorded with a PerkinElmer Spectrum 100 spectrophotometer. ICP–OES data was obtained using a PERKIN ELMER OPTIMA 5300 DV ICP–OES device.

### Preparation of the Ni–chitosan nanocatalyst

NiCl_2_·6H_2_O (500 mg) was added slowly under continuous stirring to a suspension of chitosan (5 g) in 100 mL of water. An ammonia solution was used to adjust the pH value of the mixture to 9. The solution was further stirred continuously overnight at room temperature. After that, the green catalyst was separated by filtration and dried under vacuum at 60 °C. The synthesized pure Ni–chitosan nanocatalyst was characterized by powder FTIR and XRD spectroscopy, TEM, FESEM, and EDX analysis.

### Preparation of 1,4-dihydropyridine-2,3-dicarboxylate derivatives

A primary amine **1** (1 mmol), a dialkyl but-2-ynedioate **2** (1 mmol), a cinnamaldehyde **3** (1 mmol), and 30 mg of the Ni–chitosan nanocatalyst were added to EtOH (5 mL), and the reaction mixture was then subjected to ultrasonic irradiation for 10 min. After completion of the reaction (as monitored by the disappearance of the starting materials via thin-layer chromatography), ethyl acetate (5 mL) was added to the mixture, and the solid catalyst was separated from the mixture by an external magnet. The recovered catalyst was washed with water and acetone, dried in a desiccator, and stored for a consecutive reaction. The crude products were obtained by evaporation of the solvent in a rotary evaporator and purification of the residue via silica gel (100–200 mesh) column chromatography using ethyl acetate/petroleum ether (bp 60–80 °C) as eluent.

## Supporting Information

File 1 Characterization data and copies of spectra.

File 2 CIF file for compound **4a**.

## References

[1] Zaera F (2013). Chem Soc Rev.

[2] Climent M J, Corma A, Iborra S, Sabater M J (2014). ACS Catal.

[3] Zhang L-R, Zhao J, Li M, Ni H-T, Zhang J-L, Feng X-M, Ma Y-W, Fan Q-L, Wang X-Z, Hu Z (2012). New J Chem.

[4] Nimkar A, Ramana M M V, Betkar R, Ranade P, Mundhe B (2016). New J Chem.

[5] Hajipour A R, Abolfathi P, Mohammadsaleh F (2016). RSC Adv.

[6] Magné V, Garnier T, Danel M, Pale P, Chassaing S (2015). Org Lett.

[7] Karami K, Moghadam Z K, Hosseini-Kharat M (2014). Catal Commun.

[8] Cornelio B, Rance G A, Laronze-Cochard M, Fontana A, Sapi J, Khlobystov A N (2013). J Mater Chem A.

[9] Mendes A A, de Castro P C d H F, Giordano R D L C (2011). Quim Nova.

[10] Guibal E (2005). Prog Polym Sci.

[11] Bernkop-Schnürch A, Dünnhaupt S (2012). Eur J Pharm Biopharm.

[12] Kim Y S, Milner J A (2005). J Nutr Biochem.

[13] Yong S K, Shrivastava M, Srivastava P, Kunhikrishnan A, Bolan N (2015). Rev Environ Contam Toxicol.

[14] López-Cruz A, Barrera C, Calero-DdelC V L, Rinaldi C (2009). J Mater Chem.

[15] Shahidi F, Arachchi J K V, Jeon Y-J (1999). Trends Food Sci Technol.

[16] El Ghaouth A, Arul J, Grenier J, Asselin A (1992). Phytopathology.

[17] Caetano C S, Caiado M, Farinha J, Fonseca I M, Ramos A M, Vital J, Castanheiro J E (2013). Chem Eng J.

[18] Hajipour A R, Rezaei F, Khorsandi Z (2017). Green Chem.

[19] Macquarrie D J, Hardy J J E (2005). Ind Eng Chem Res.

[20] Xiao L-F, Li F-W, Xia C-G (2005). Appl Catal, A.

[21] Pizzuti L, Franco M S F, Flores A F C, Quina F H, Pereira C M P, Mishra N K (2012). Recent Advances in the Ultrasound-Assisted Synthesis of Azoles. Green Chemistry – Environmentally Benign Approaches.

[22] Cella R, Stefani H A (2009). Tetrahedron.

[23] Ray S, Manna P, Mukhopadhyay C (2015). Ultrason Sonochem.

[24] Huang Q, Li Y, Sheng C, Dou Y, Zheng M, Zhu Z, Wang J (2015). J Hypertens.

[25] Bossert F, Vater W (1989). Med Res Rev.

[26] Das B, Suneel K, Venkateswarlu K, Ravikanth B (2008). Chem Pharm Bull.

[27] Reddy V H, Kumari A K, Reddy G M, Reddy Y V R, Garcia J R, Zyryanov G V, Reddy N B, Rammohan A (2019). Chem Heterocycl Compd.

[28] Wang X, Du Y, Fan L, Liu H, Hu Y (2005). Polym Bull.

[29] Khan A T, Khan M M (2011). Tetrahedron Lett.

[30] Ugarriza I, Uria U, Carrillo L, Vicario J L, Reyes E (2014). Chem – Eur J.

[31] Jiang J, Yu J, Sun X-X, Rao Q-Q, Gong L-Z (2008). Angew Chem, Int Ed.

[32] Wan J-P, Gan S-F, Sun G-L, Pan Y-J (2009). J Org Chem.

[33] Kantam M L, Ramani T, Chakrapani L, Choudary B M (2009). Catal Commun.

