# Supplementary material for: Green synthesis of C5–C6-unsubstituted 1,4-DHP scaffolds using an efficient Ni–chitosan nanocatalyst under ultrasonic conditions
Source: Beilstein J Org Chem. 2022 Jan 25;18:133–42. doi: 10.3762/bjoc.18.14 (PMC8805040; doi:10.3762/bjoc.18.14)
Supplement: File 1 — Characterization data and copies of spectra. [file Beilstein_J_Org_Chem-18-133-s001.pdf]

BEILSTEIN JOURNAL OF ORGANIC CHEMISTRY

Supporting Information

for

Green synthesis of C5–C6-unsubstituted 1,4-DHP scaffolds using an efficient Ni–chitosan nanocatalyst under ultrasonic conditions

Soumyadip Basu, Sauvik Chatterjee, Suman Ray, Suvendu Maity, Prasanta Ghosh, Asim Bhaumik and Chhanda Mukhopadhyay

Beilstein J. Org. Chem. **2022,** *18,* 133–142. doi:10.3762/bjoc.18.14

Characterization data and copies of spectra

License and Terms: This is a supporting information file under the terms of the Creative Commons Attribution License (https://creativecommons.org/licenses/by/4.0). Please note that the reuse, redistribution and reproduction in particular requires that the author(s) and source are credited and that individual graphics may be subject to special legal provisions.
The license is subject to the *Beilstein Journal of Organic Chemistry* terms and conditions: (https://www.beilstein-journals.org/bjoc/terms)

Table of contents

1. Characterization data of all the synthesized compounds S2

2. ^{1}H NMR and ^{13}C NMR spectra for **4a–q** and **4fA** S7

3. Crude mass spectra of compound **4k** and **4m** S25

Characterization data of synthesized compounds:

Dimethyl 4-phenyl-1-(*p*-tolyl)-1,4-dihydropyridine-2,3-dicarboxylate (4a, yield: 88%) Yellowish solid; R_f : 0.40 (25% ethyl acetate in petroleum ether); M.p: 132 ^{0}C;δ_H (300 MHz, $CDCl_3$): 7.42-7.34 (m, 4H), 7.27-7.19 (m, 5H), 6.19 (d, *J* = 7.5 Hz, 1H), 5.06 (dd, *J* = 5.4 Hz, 1H), 4.60(d, *J* = 5.1 Hz, 1H), 3.59 (s, 3H), 3.56 (s, 3H), 2.37 (s, 3H); δ_C (75 MHz, $CDCl_3$): 167.2, 164.8, 147.1, 143.5, 139.4, 137.8, 129.9, 128.5, 127.5, 126.5, 126.4, 108.2, 100.8, 52.3, 51.4, 38.4, 21.0; HRMS: calcd for $C_{22}H_{21}NO_4$ [M + H] 364.1549, found 364.1586.

Diethyl 4-phenyl-1-(*p*-tolyl)-1,4-dihydropyridine-2,3-dicarboxylate (4b, yield: 87%). Yellowish solid; R_f : 0.34 (25% ethyl acetate in petroleum ether); M.p: 136^{0}C;δ_H (300 MHz, $CDCl_3$): 7.21-7.10 (m, 4H), 7.04-6.96 (m, 5H), 5.95 (d, *J* = 7.8 Hz, 1H), 4.83-4.80 (m, 1H), 4.39 (d, *J* = 5.1 Hz, 1H), 3.82-3.76 (m, 4H), 2.13 (s, 3H), 0.89 (t,*J* = 7.2Hz, 3H), 0.78 (t, *J* = 7.2 Hz, 3H); δ_C (75 MHz, $CDCl_3$): 166.6, 164.2, 147.3, 143.4, 139.5, 137.8, 129.7, 128.6, 128.4, 127.7, 126.8, 126.4, 108.0, 100.9, 61.4, 60.0, 38.6, 21.0, 13.9, 13.5; HRMS: calcd for $C_{24}H_{25}NO_4$, [M + H] 392.1862, found 392.1815.

Dimethyl 1-(4-methoxyphenyl)-4-phenyl-1,4-dihydropyridine-2,3-dicarboxylate (4c, yield: 89%). Light yellowish solid; R_f : 0.36 (25% ethyl acetate in petroleum ether); M.p: 138 ^{0}C;δ_H (300 MHz, $CDCl_3$): 7.22-7.14 (m, 5H), 7.03 (d, *J* = 8.4 Hz, 2H), 6.70 (d, *J* = 8.7Hz, 2H), 5.94 (d, *J* = 7.8 Hz, 1H), 4.85 (dd, *J* = 5.4 Hz, 1H), 4.41 (d, *J* = 5.4Hz, 1H), 3.62 (s, 3H), 3.41 (s, 3H), 3.39 (s, 3H); δ_C (75 MHz, $CDCl_3$): 167.2, 164.8, 159.0, 147.2, 143.8, 134.7, 128.9, 128.3, 127.5, 126.5, 114.3, 108.9, 100.3, 55.4, 52.3, 51.3, 38.4.

Diethyl 1-(2,5-dichlorophenyl)-4-phenyl-1,4-dihydropyridine-2,3-dicarboxylate (4d, yield: 80%). Yellowish solid; R_f : 0.37 (25% ethyl acetate in petroleum ether); M.p: 142 ^{0}C;δ_H (300 MHz, $CDCl_3$): 7.41-7.33 (m, 5H), 7.28-7.21 (m, 3H), 6.18 (d, *J* = 7.8 Hz, 1H), 5.07 (dd,

J = 5.1 Hz, 1H), 4.61 (d, *J* = 5.1 Hz, 1H), 4.06-3.99 (m, 4H), 1.12 (t,*J* = 7.2 Hz, 3H), 1.04 (t,*J* = 7.2 Hz, 3H);δ_C (75 MHz, $CDCl_3$): 166.4, 164.0, 146.9, 142.7, 140.6, 133.6, 129.4, 128.9, 128.3, 128.1, 127.4, 126.6, 125.9, 108.6, 102.1, 61.7, 60.2, 38.6, 13.9, 13.5.

Dimethyl 1-(4-nitrophenyl)-4-phenyl-1,4-dihydropyridine-2,3-dicarboxylate (4e, yield: 78%). Yellowish solid; R_f : 0.31 (25% ethyl acetate in petroleum ether); M.p: 146 ^{0}C;δ_H (300 MHz, $CDCl_3$): 8.05 (d, *J* = 8.4 Hz, 2H), 7.37-7.27 (m, 5H),7.09 (d, *J* = 8.1 Hz, 2H), 6.49 (d, *J* = 9.6 Hz, 1H), 5.79-5.74 (m, 1H), 5.32 (d, *J* = 5.7 Hz, 1H), 3.65 (s, 6H); δ_C (75 MHz, $CDCl_3$): 165.4, 164.6, 150.0, 144.6, 140.9, 129.2, 128.9, 128.3, 124.9, 124.8, 122.7, 120.4, 119.5, 111.9, 100.8, 62.9, 52.9, 51.9, 38.4.

Diethyl 1-benzyl-4-phenyl-1,4-dihydropyridine-2,3-dicarboxylate (4f, yield: 82%). White solid; R_f : 0.44 (25% ethyl acetate in petroleum ether); M.p: 134 ^{0}C;δ_H(300 MHz, $CDCl_3$): 7.30-7.27 (m, 7H), 7.19-7.18 (m, 3H), 5.81 (d, *J* = 7.5Hz, 1H), 4.91 (dd, *J* = 5.1 Hz, 1H), 4.48 (d, *J* = 5.1 Hz, 1H), 4.35 (d, *J* = 8.4 Hz, 2H), 4.22 (q, *J* = 7.2 Hz, 2H), 3.93 (d, *J* = 7.2 Hz, 2H),1.15 (t,*J* = 7.2 Hz, 3H), 1.02 (t,*J* = 7.2 Hz, 3H); δ_C (75 MHz, $CDCl_3$): 166.8, 165.2, 147.3, 143.9, 136.5, 128.6, 128.4, 128.2, 127.6, 127.4, 127.0, 126.2, 109.1, 99.8, 61.9, 59.9, 54.4, 38.6, 13.9, 13.6.

Dimethyl 1-benzyl-4-phenyl-1,4-dihydropyridine-2,3-dicarboxylate (4g, yield: 81%). Light yellowish solid; R_f : 0.42 (25% ethyl acetate in petroleum ether); M.p: 136 ^{0}C;δ_H (300 MHz, $CDCl_3$): 7.41-7.34 (m, 7H), 7.31-7.26 (m, 3H), 5.92 (d, *J* = 7.8 Hz, 1H), 5.01 (dd, *J* = 5.4 Hz, 1H), 4.57 (d, *J* = 5.4 Hz, 1H), 4.43 (d, *J* = 9.9 Hz, 2H), 3.87 (s, 3H),3.60 (s, 3H); δ_C (75 MHz, $CDCl_3$): 167.4, 165.7, 147.1, 144.0, 136.4, 128.8, 128.4, 128.0, 127.6, 127.0, 126.6, 126.4, 109.3, 99.8, 54.7, 52.8, 51.3, 38.4;

Dimethyl 1-(3-nitrophenyl)-4-phenyl-1,4-dihydropyridine-2,3-dicarboxylate (4h, yield: 84%). Yellowish solid; R_f : 0.32 (25% ethyl acetate in petroleum ether); M.p: 140 ^{0}C; δ_H (300 MHz, $CDCl_3$): 7.99-7.90(m, 2H), 7.45 (d, *J* = 7.8 Hz, 1H), 7.41-7.28 (m, 6H), 6.52 (d, *J* = 7.5Hz, 1H), 5.64 (dd, *J* = 6.0Hz, 1H), 5.22 (d, *J* = 5.1 Hz, 1H), 3.85 (s, 3H),3.51 (s, 3H); δ_C (75 MHz, $CDCl_3$): 167.8, 165.5, 148.5, 145.2, 141.3, 139.1, 131.3,128.7, 128.3, 127.5, 125.4, 120.9, 120.3, 119.1, 118.2, 108.2, 52.7, 51.4, 39.6.

Diethyl 1-benzyl-4-(4-nitrophenyl)-1,4-dihydropyridine-2,3-dicarboxylate (4i, yield: 86%). Yellowish solid; R_f : 0.35 (25% ethyl acetate in petroleum ether); M.p: 182-142 ^{0}C; δ_H (400 MHz, $CDCl_3$): 8.11(d, *J* = 7.2Hz, 2H), 7.39-7.26 (m, 7H), 5.95 (d, *J* = 7.6Hz, 1H), 4.91 (dd, *J* = 5.2Hz, 1H), 4.69 (d, *J* = 5.2Hz, 1H), 4.43(ABq, *J* = 15.6Hz, 2H), 4.32-4.28(m, 2H),4.02-4.00 (m, 2H),1.24 (t,*J* = 7.2 Hz, 3H), 1.09 (t,*J* = 7.2 Hz, 3H); δ_C (100 MHz, $CDCl_3$): 166.4, 164.9, 154.3, 146.7, 144.7, 136.4, 129.0,128.8, 128.3, 127.7, 123.9, 123.0, 107.9, 99.0, 62.4, 60.5, 54.9, 39.0, 14.2, 13.8.

Dimethyl 1-(4-methoxyphenyl)-4-(4-nitrophenyl)-1,4-dihydropyridine-2,3-dicarboxylate (4j, yield: 87%). Yellowish solid; R_f : 0.37 (25% ethyl acetate in petroleum ether); M.p: 140 ^{0}C; δ_H (400 MHz, $CDCl_3$): 8.20 (d, *J* = 8.8 Hz, 2H), 7.52 (d, *J* = 8.4 Hz, 2H), 7.19 (d, *J* = 8.8Hz, 2H), 6.88 (d, *J* = 8.7 Hz, 2H), 6.16(d, *J* = 7.6Hz, 1H), 4.97 (dd, *J* = 5.2Hz, 1H), 4.73 (d, *J* = 5.2Hz, 1H), 3.80 (s, 3H), 3.54 (s, 3H), 3.52 (s, 3H); δ_C (100 MHz, $CDCl_3$): 166.7, 164.3, 159.2, 154.0, 146.5, 144.4, 134.1, 129.1, 128.3, 123.9, 123.7, 114.4, 106.5, 98.9, 55.4, 52.9, 51.6, 38.5.

Dimethyl 1,4-bis(4-methoxyphenyl)-1,4-dihydropyridine-2,3-dicarboxylate (4k, yield: 82%). Light yellowish solid; R_f : 0.37 (25% ethyl acetate in petroleum ether); M.p: 138 ^{0}C; δ_H (400 MHz, $CDCl_3$): 7.30 (d, *J* = 8.4 Hz, 2H), 7.20 (d, *J* = 6.8 Hz, 2H), 6.89-6.86 (m, 4H), 6.11(d, *J* = 8.0Hz, 1H), 5.01 (dd, *J* = 5.2 Hz, 1H), 4.52 (d, *J* = 5.2 Hz, 1H), 3.81 (s, 3H), 3.80

(s, 3H), 3.57 (s, 3H), 3.54 (s, 3H); δ_C (100 MHz, $CDCl_3$): 167.3, 164.9, 158.9, 158.2, 143.5, 139.7, 134.7, 128.5, 128.3, 127.9, 114.1, 113.8, 108.2, 100.5, 55.3, 55.2,52.4, 51.4, 37.4; HRMS: calcd for $C_{23}H_{23}NO_5$ [M + H] 410.1604, found 410.1658.

Dimethyl 1-benzyl-4-(4-nitrophenyl)-1,4-dihydropyridine-2,3-dicarboxylate (4l, yield: 86%). Yellowish solid; R_f : 0.35 (25% ethyl acetate in petroleum ether); M.p: 148 ^{0}C;δ_H (400 MHz, $CDCl_3$): 8.11 (d, J = 6.8 Hz, 2H), 7.41-7.29 (m, 7H), 5.95 (d, J = 7.6Hz, 1H), 4.93 (dd, J = 5.2Hz, 1H), 4.66 (d, J = 5.4 Hz, 1H), 4.41(ABq, J = 15.6 Hz, 2H), 3.85(s, 3H),3.56 (s, 3H); δ_C (100 MHz, $CDCl_3$): 159.8, 158.3, 146.9, 139.5, 137.5, 129.7, 121.8, 121.3, 121.0, 120.8, 120.8, 116.8, 100.9, 91.6, 47.9, 46.0, 44.6, 31.5.

Dimethyl 1-benzyl-4-(4-methoxyphenyl)-1,4-dihydropyridine-2,3-dicarboxylate (4m, yield: 76%). Light yellowish solid; R_f : 0.37 (25% ethyl acetate in petroleum ether); M.p: 136 ^{0}C;δ_H (400 MHz, $CDCl_3$): 7.32-7.25 (m, 4H), 7.23-7.19 (m, 3H), 6.83 (d, J = 6.8 Hz, 2H), 6.37 (d, J = 8.0 Hz, 1H), 5.02-5.00 (m, 1H), 4.83 (d, J = 4.8 Hz, 1H), 4.08 (ABq, J = 15.6 Hz, 2H), 3.82 (s, 3H), 3.73 (s, 3H), 3.65 (s, 3H); δ_C (100 MHz, $CDCl_3$): 166.1, 165.7, 159.7, 148.8, 135.2, 134.1, 128.6, 128.1, 127.9, 119.7, 115.3, 114.3, 113.7, 95.1, 55.2, 53.5, 53.0, 51.2, 38.1; HRMS: calcd for $C_{23}H_{23}NO_5$ [M + H] 394.1654, found 394.1678.

Diethyl 1-benzyl-4-(4-methoxyphenyl)-1,4-dihydropyridine-2,3-dicarboxylate (4n, yield: 79%). Yellowish solid; R_f : 0.39 (25% ethyl acetate in petroleum ether); M.p: 138 ^{0}C;δ_H (400 MHz, $CDCl_3$): 7.37-7.31 (m, 5H), 7.15 (d, J = 6.4 Hz, 2H), 6.79 (d, J = 6.8 Hz, 2H), 5.88 (d, J = 8.0 Hz, 1H), 4.93 (dd, J = 5.2 Hz, 1H), 4.48 (d, J = 5.2 Hz, 1H), 4.41 (d, J = 14.0 Hz, 2H), 4.17 (q, J = 7.2 Hz, 2H), 4.01 (q, J = 7.2 Hz, 2H), 3.78 (s, 3H), 1.21 (t, J = 7.2 Hz, 3H), 1.11 (t,J = 7.2 Hz, 3H); δ_C (100 MHz, $CDCl_3$): 166.9, 165.3, 158.1, 143.6, 139.9, 136.6, 128.7, 128.1, 127.9, 127.4, 126.9, 113.5, 109.3, 100.1, 62.0, 59.7, 55.2, 53.3, 37.7, 14.0, 13.7.

Dimethyl 1-(4-methoxybenzyl)-4-phenyl-1,4-dihydropyridine-2,3-dicarboxylate (4o, yield: 82%). Yellowish solid; R_f : 0.34 (25% ethyl acetate in petroleum ether); M.p: 130 ^{0}C; δ_H (400 MHz, $CDCl_3$): 7.45-7.29 (m, 3H), 7.23-7.19 (m, 4H), 6.89 (d, *J* = 6.8 Hz, 2H), 5.87 (d, *J* = 7.6 Hz, 1H), 4.97-4.94 (m, 1H), 4.51 (d, *J* = 5.2 Hz, 1H), 4.35-4.31 (m, 2H), 3.88 (s, 3H), 3.82 (s, 3H), 3.55 (s, 3H); δ_C (100 MHz, $CDCl_3$): 167.4, 165.7, 159.2, 147.0, 143.9, 128.6, 128.3, 127.9, 127.2, 126.7, 126.3, 113.8, 109.2, 55.1, 54.1, 52.6, 51.2, 38.3.

Dimethyl 1-(4-methoxybenzyl)-4-(4-methoxyphenyl)-1,4-dihydropyridine-2,3-dicarbox-ylate (4p, yield: 82%). Yellowish solid; R_f : 0.37 (25% ethyl acetate in petroleum ether); M.p: 134^{0}C; δ_H (400 MHz, $CDCl_3$): 7.23 (d, *J* = 8.8 Hz, 2H), 7.11 (d, *J* = 8.8 Hz, 2H), 6.89 (d, *J* = 8.8 Hz, 2H), 6.79 (d, *J* = 8.8 Hz, 2H), 5.86 (d, *J* = 7.6 Hz, 1H), 5.09-5.03 (m, 1H), 4.40 (d, *J* = 5.2 Hz, 1H), 4.32 (ABq, *J* = 16 Hz, 2H), 3.90 (s, 3H), 3.84 (s, 3H), 3. 71 (s, 3H), 3.56 (s, 3H) ; δ_C (100 MHz, $CDCl_3$): 167.5, 165.9, 159.6, 158.3, 143.6, 139.7, 134.0, 128.9, 128.3, 126.8, 115.2, 114.2, 113.6, 109.5, 59.7, 55.2, 54.2, 52.8, 51.3, 37.4.

Dimethyl 1-butyl-4-phenyl-1,4-dihydropyridine-2,3-dicarboxylate (4q, yield: 76%). Light yellow solid; R_f : 0.42 (25% ethyl acetate in petroleum ether); M.p: 118 ^{0}C; δ_H (400 MHz, $CDCl_3$): 7.31-7.25 (m, 5H), 5.88 (d, *J* = 7.6 Hz, 1H), 4.98 (dd, *J* = 5.2 Hz, 1H), 4.50 (d, *J* = 5.2 Hz, 1H), 3.90 (s, 3H), 3.53 (s, 3H), 3.17 (t, *J* = 6.4 Hz, 2H), 1.66-1.58 (m, 2H), 1.35-1.27 (m, 2H), 0.92 (t, *J* = 7.2 Hz, 3H) ; δ_C (100 MHz, $CDCl_3$): 167.5, 165.7, 147.5, 144.1, 128.4, 127.4, 127.1, 126.3, 108.9, 98.7, 52.8, 51.5, 51.3, 38.3, 32.1, 19.7, 13.7.

Diethyl 2-(benzylamino)maleate (intermediate 4fA) : δ_H (300 MHz, $CDCl_3$): 8.46 (s, 1H), 7.37-7.26 (m, 5H), 5.19 (s, 1H), 4.58 (d, *J* = 6.3 Hz, 2H), 4.26-4.11 (m, 4H), 1.29-1.23 (m, 6H); δ_C (75 MHz, $CDCl_3$): 170.0, 163.6, 151.5, 138.8, 128.5, 127.2, 127.1, 88.1, 67.7, 59.1, 48.4, 14.2, 13.7.

^{1}H NMR and ^{13}C NMR spectra for 4a–q:

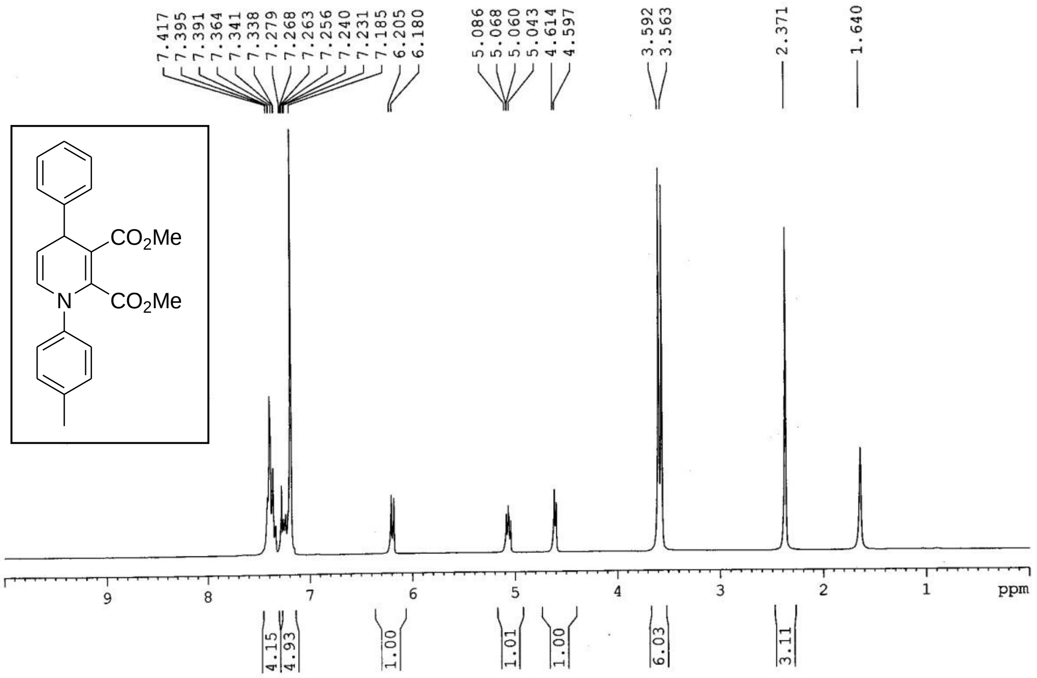

^{1}H NMR spectrum for 4a

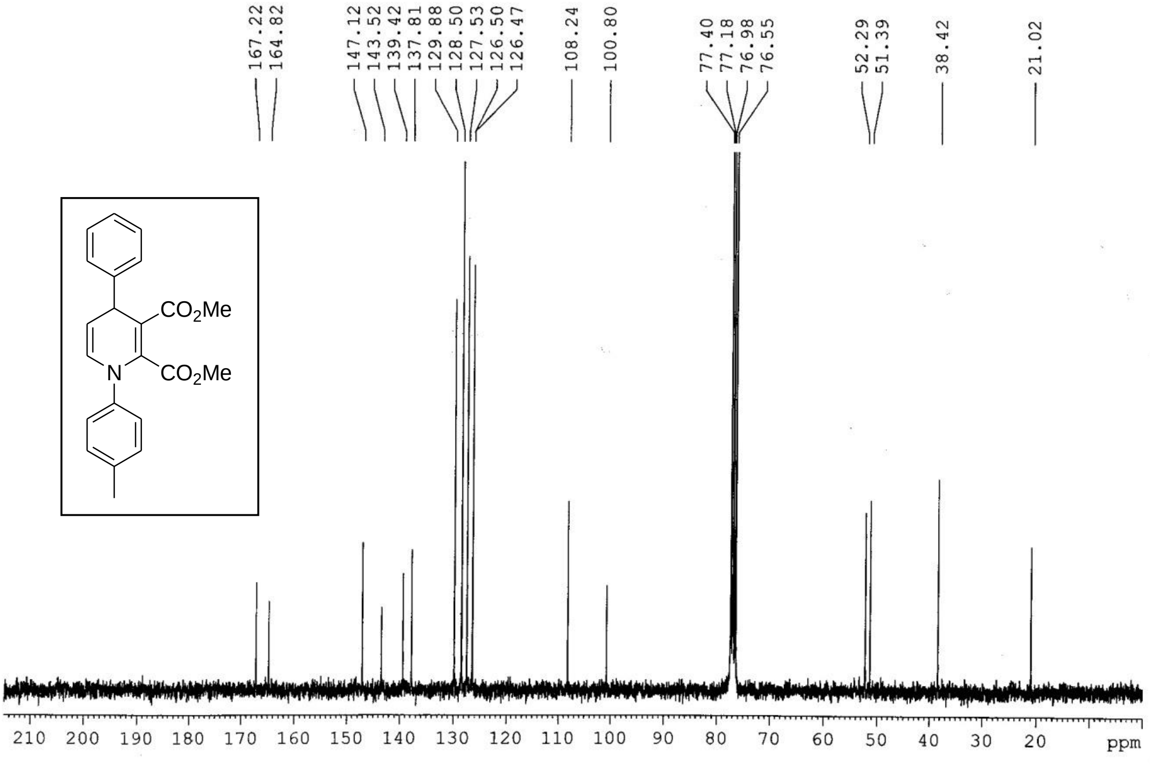

^{13}C NMR spectrum for 4a

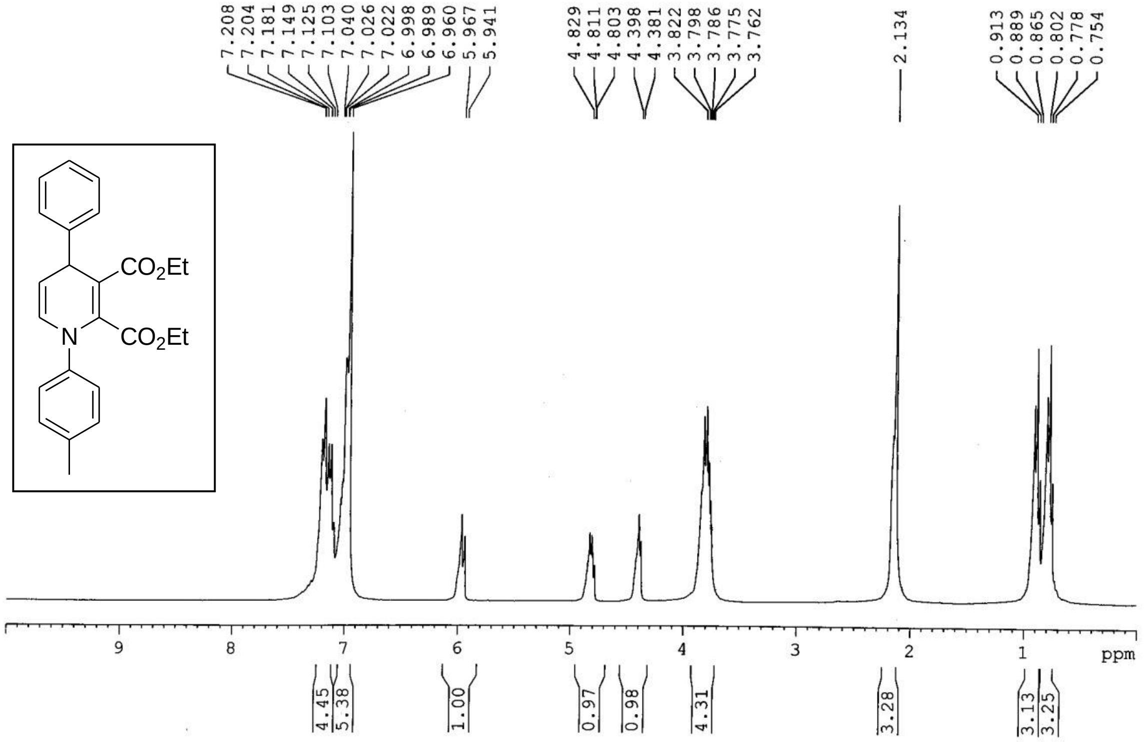

^{1}H NMR spectrum for 4b

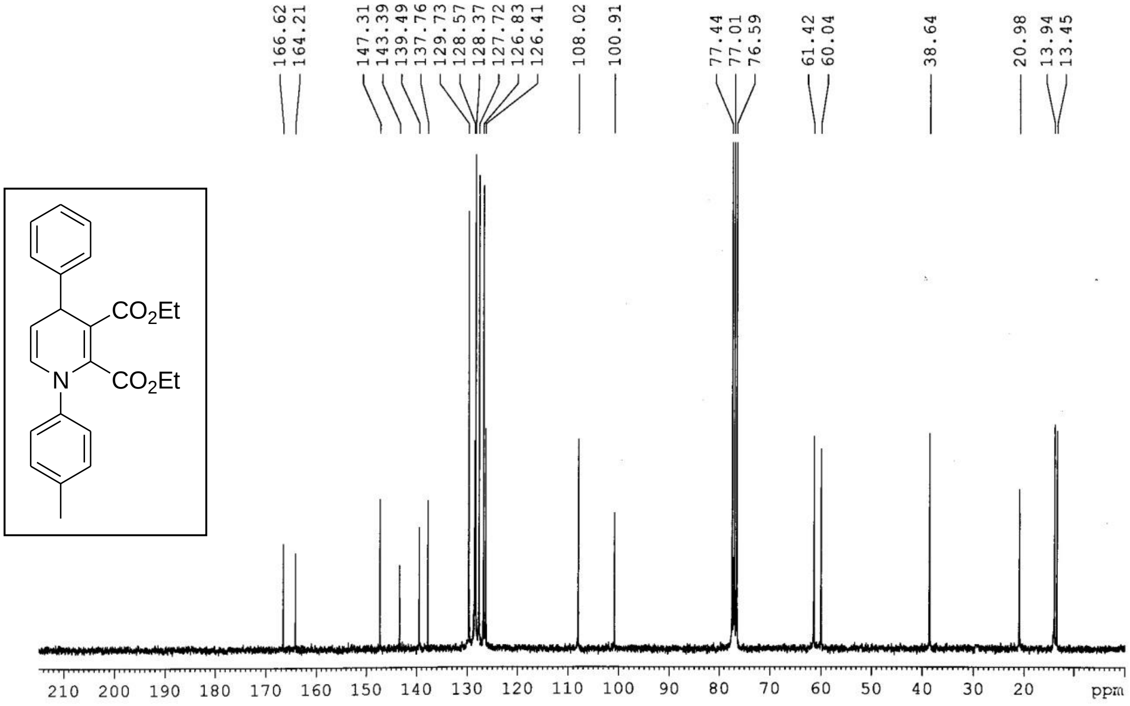

^{13}C NMR spectrum for 4b

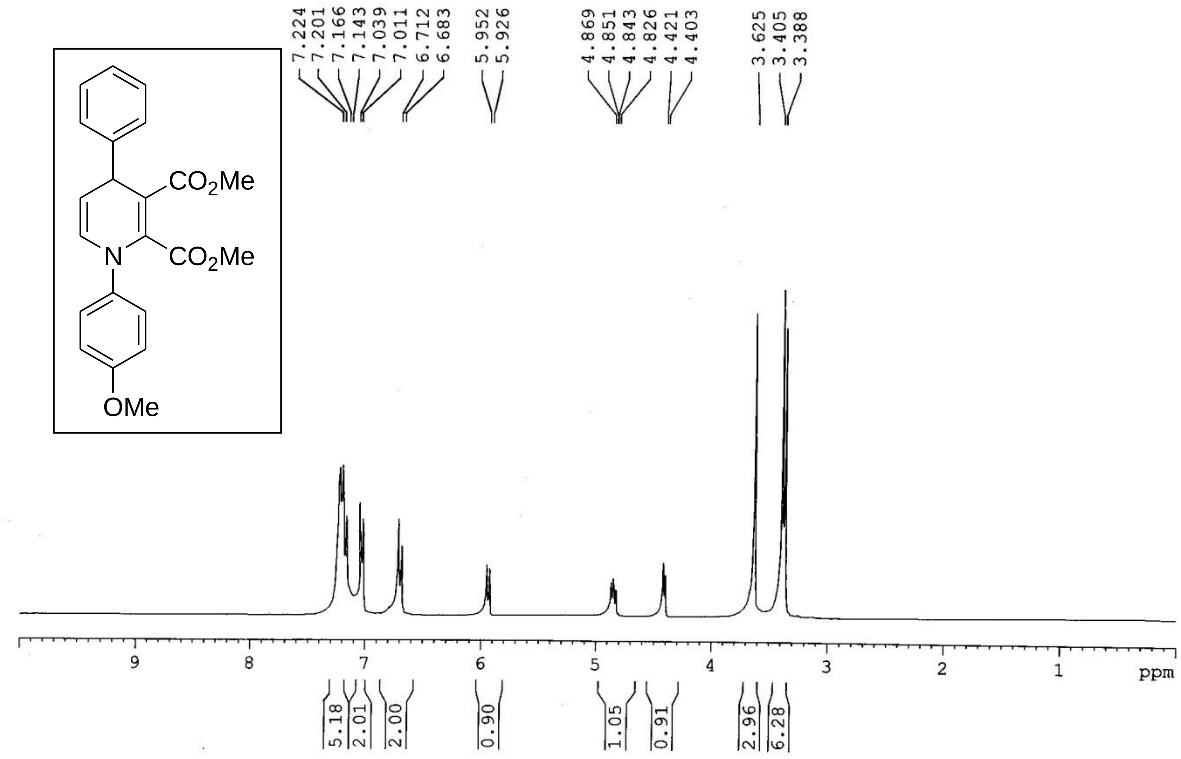

^{1}H NMR spectrum for 4c

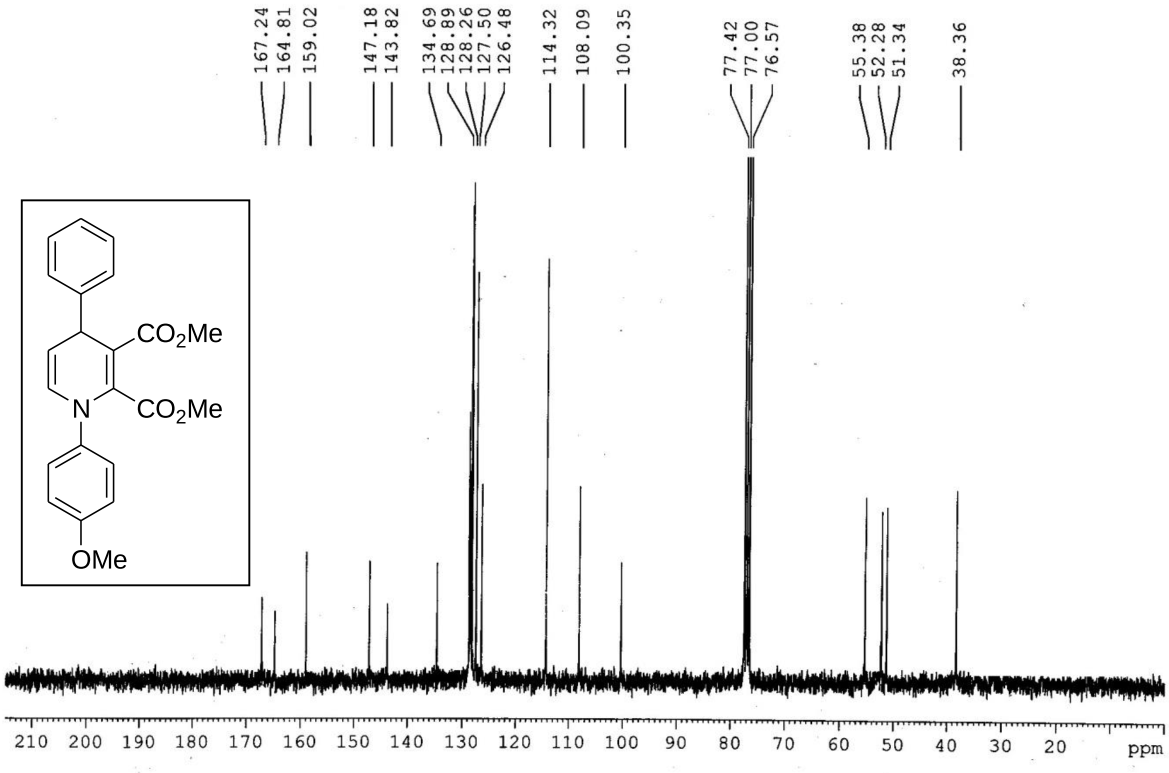

^{13}C NMR spectrum for 4c

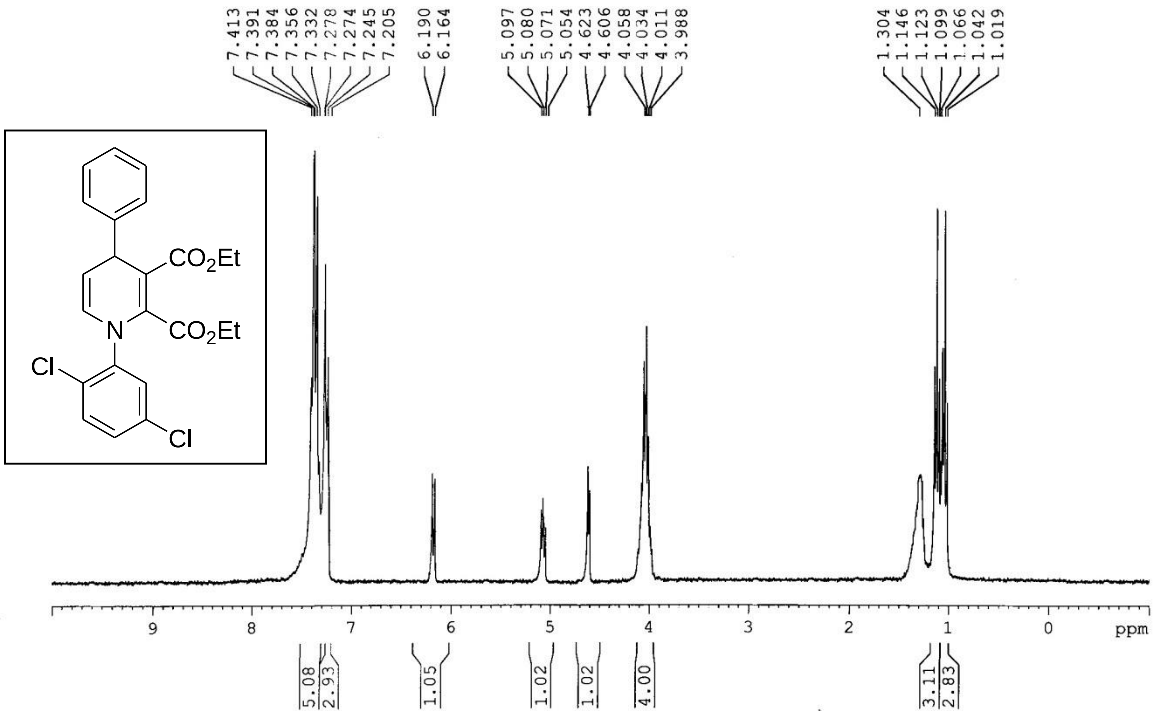

^{1}H NMR spectrum for 4d

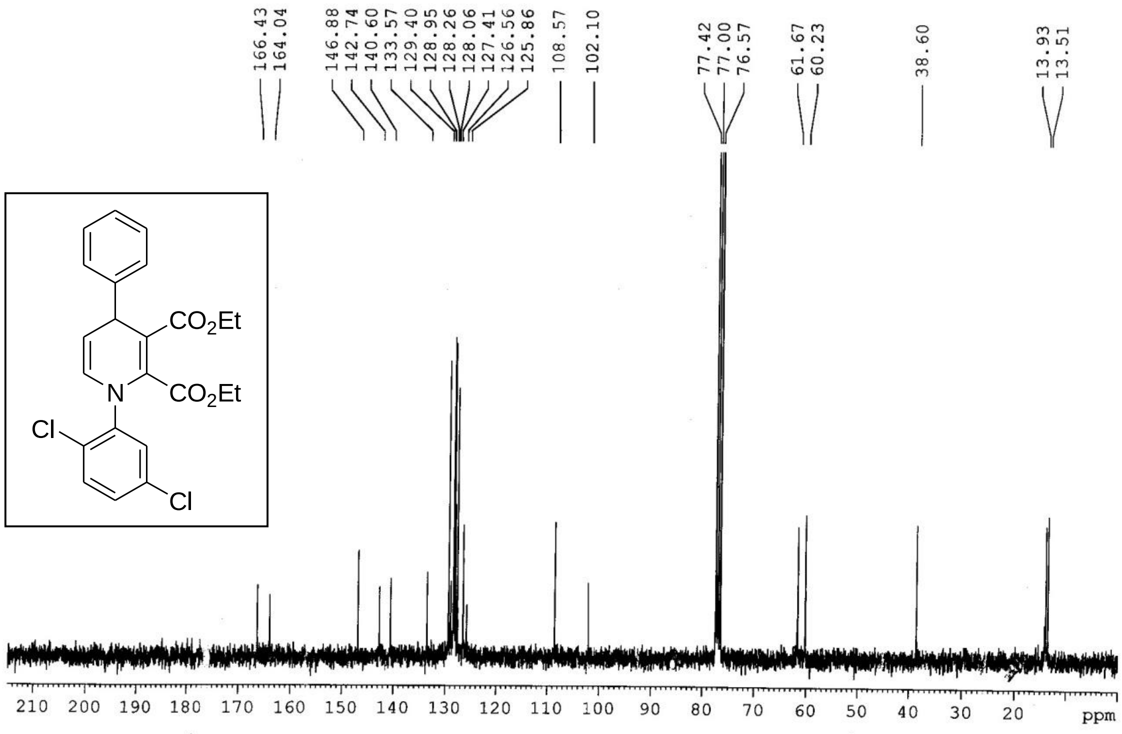

^{13}C NMR spectrum for 4d

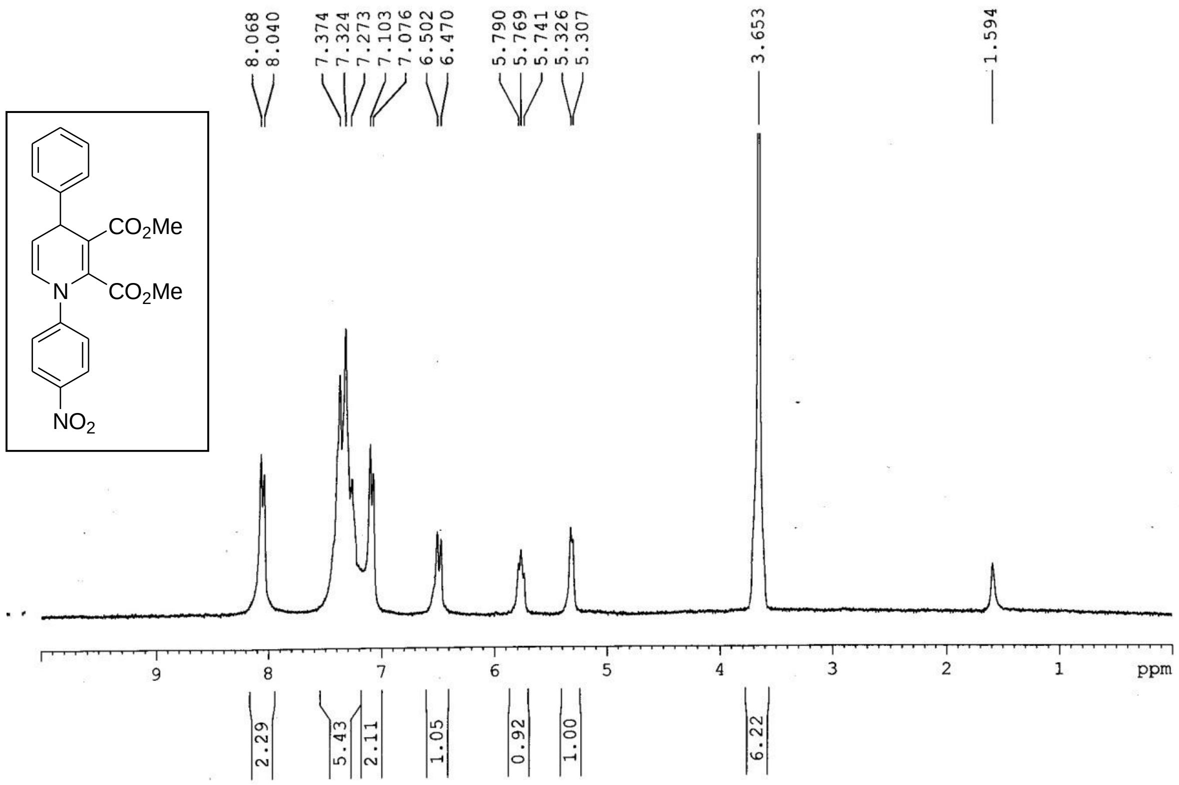

^{1}H NMR spectrum for 4e

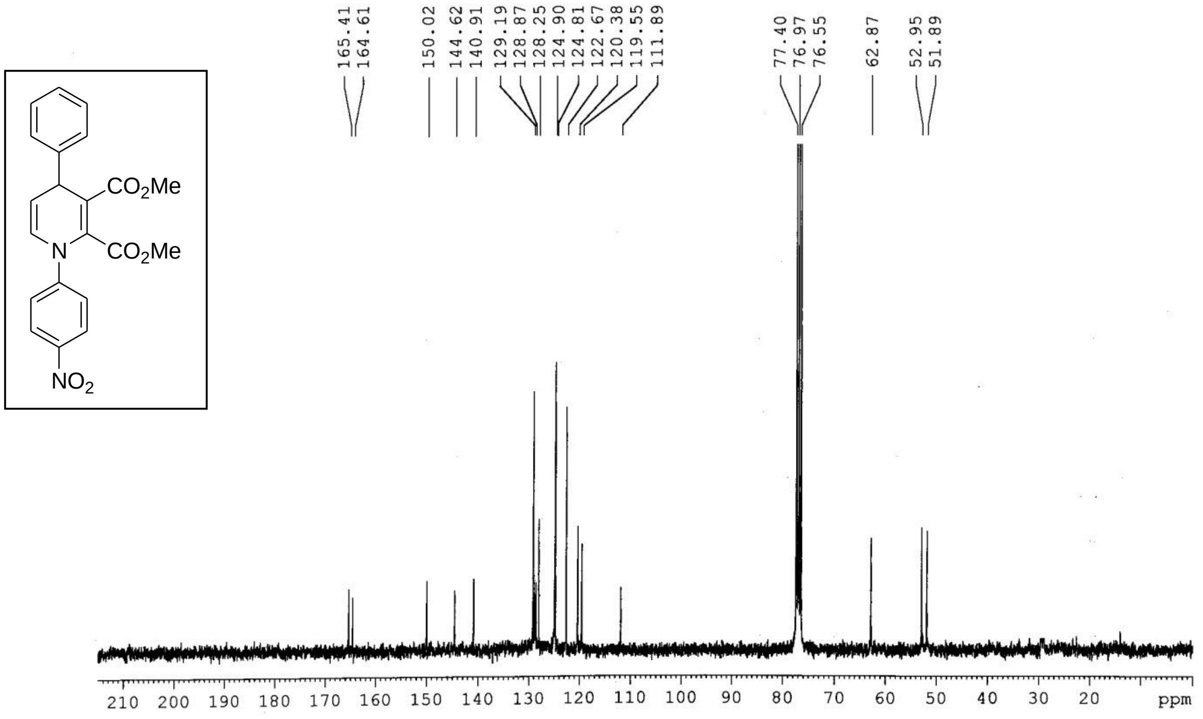

^{13}C NMR spectrum for 4e

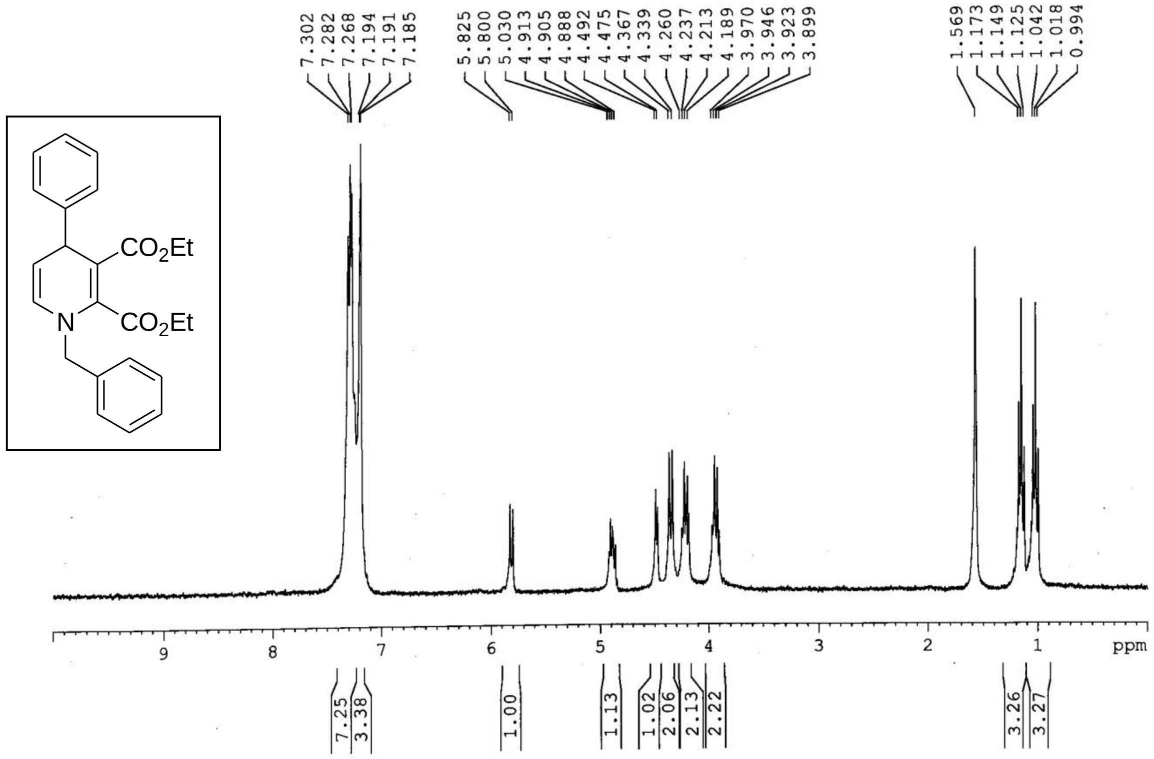

^{1}H NMR spectrum for 4f

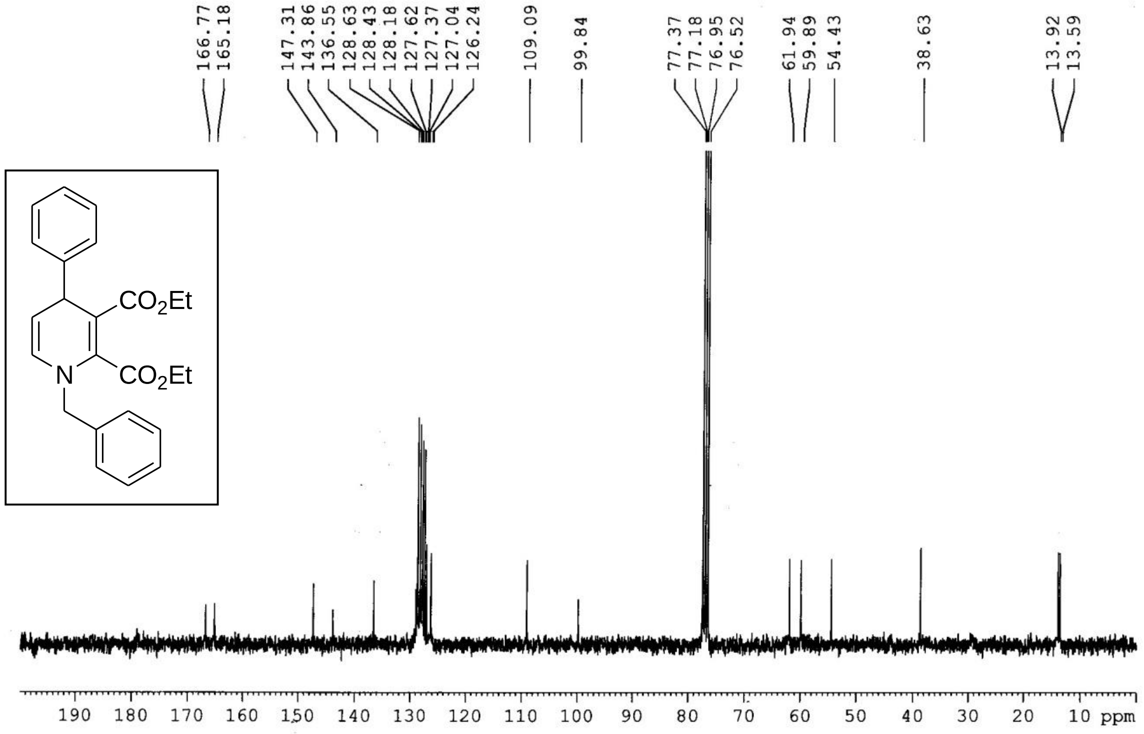

^{13}C NMR spectrum for 4f

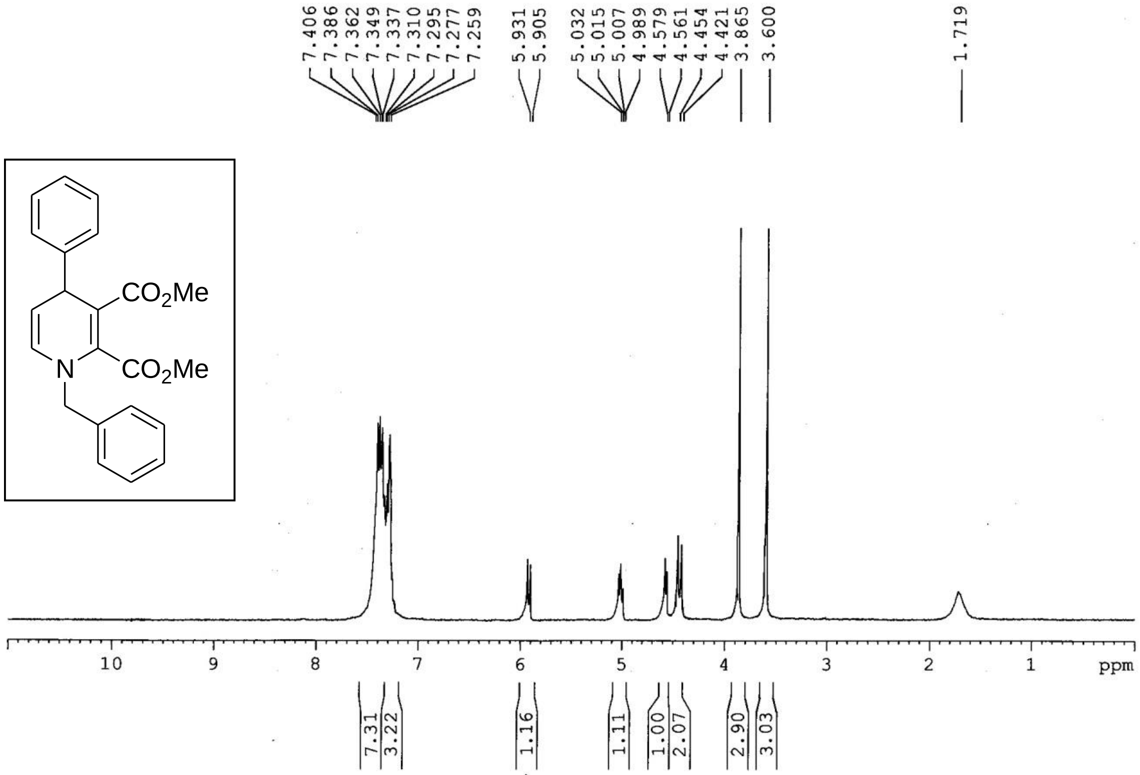

^{1}H NMR spectrum for 4g

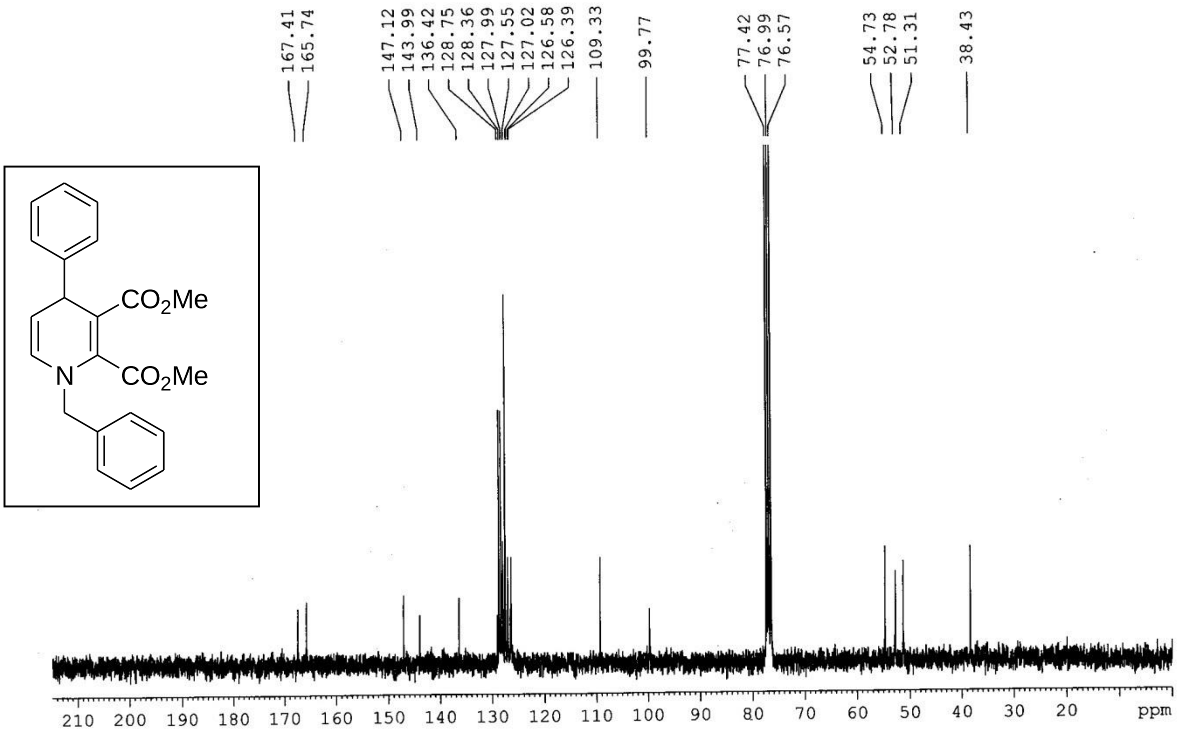

^{13}C NMR spectrum for 4g

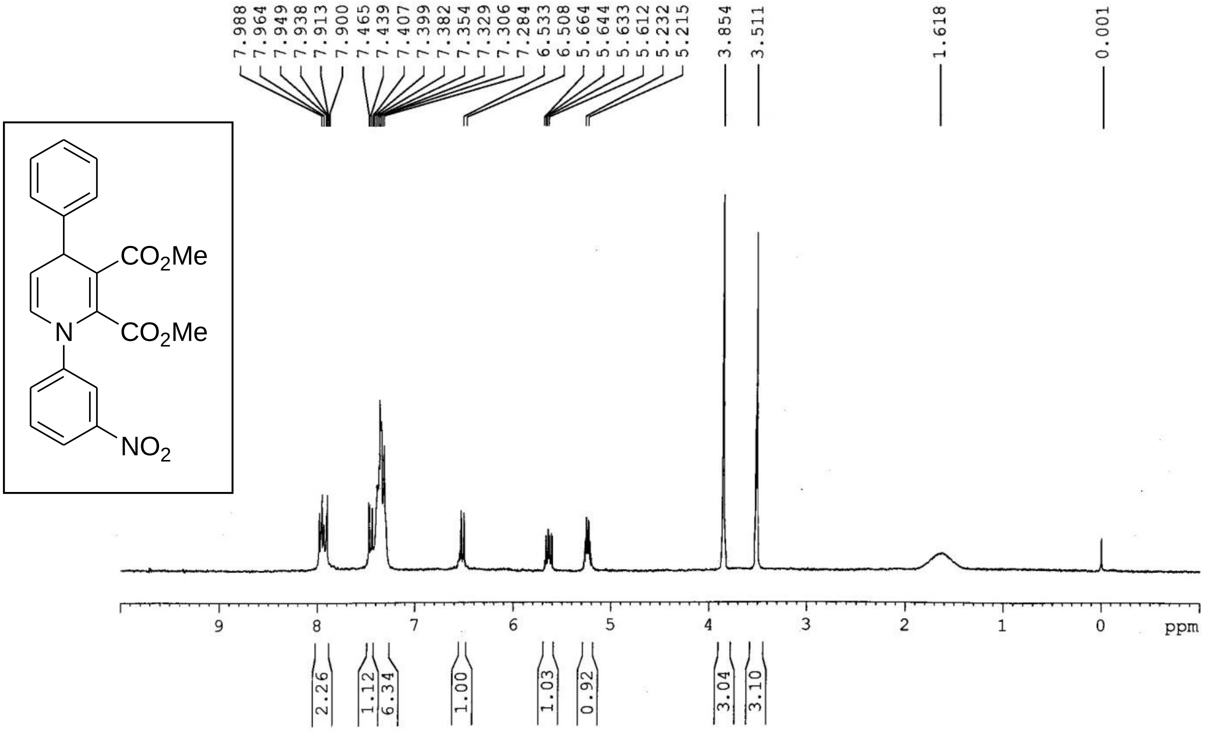

^{1}H NMR spectrum for 4h

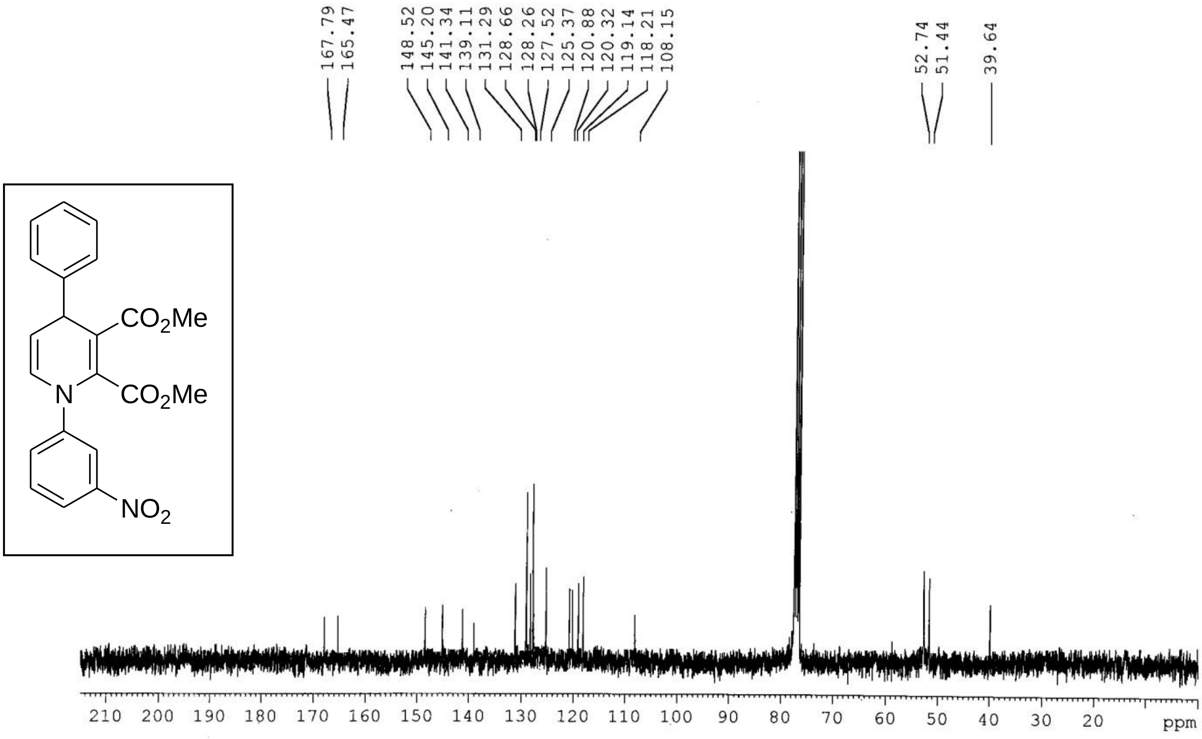

^{13}C NMR spectrum for 4h

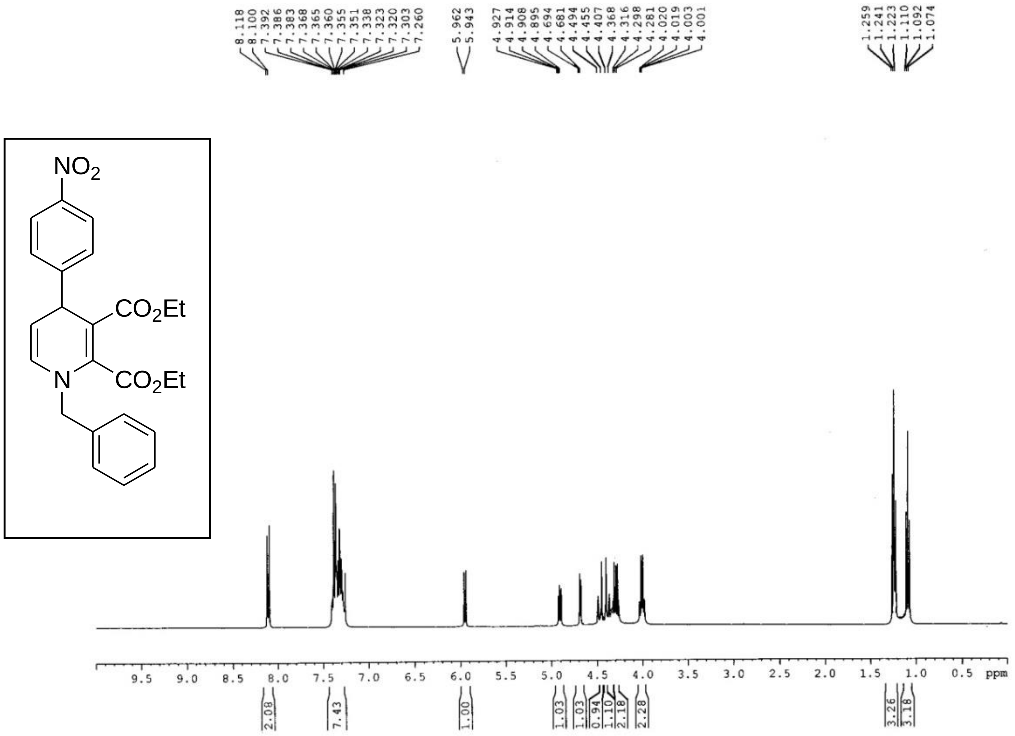

^{1}H NMR spectrum for 4i

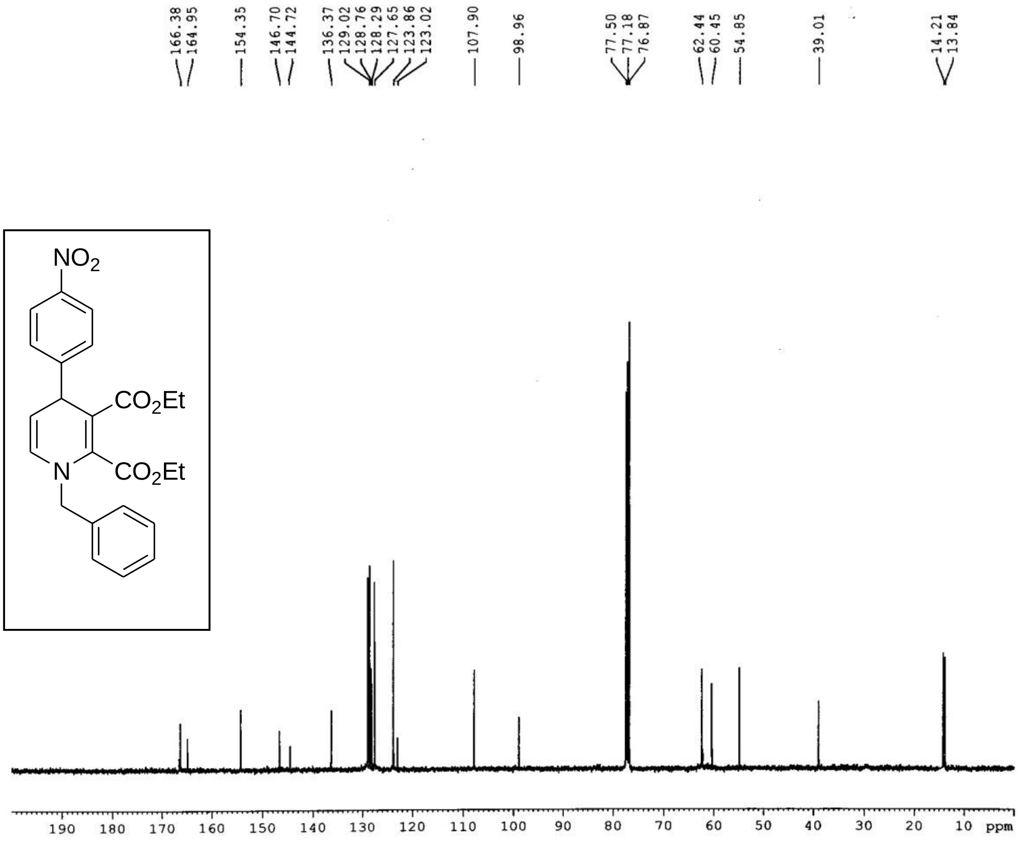

^{13}C NMR spectrum for 4i

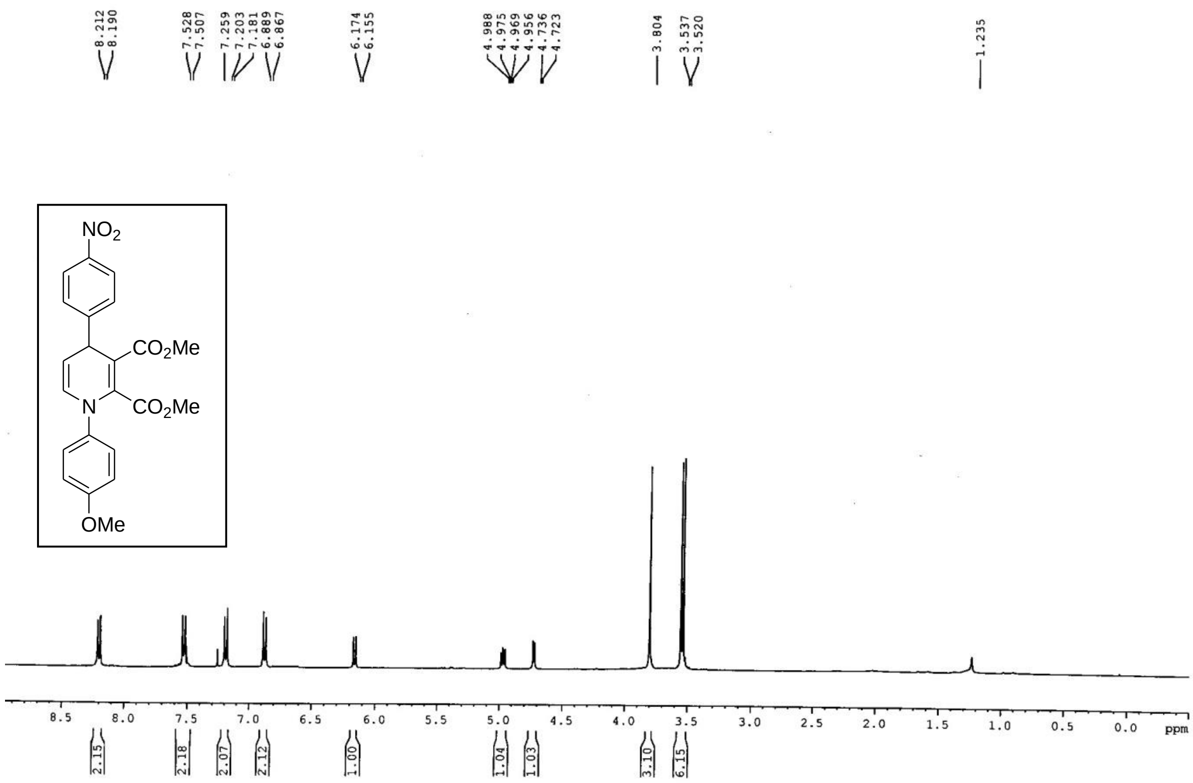

^{1}H NMR spectrum for 4j

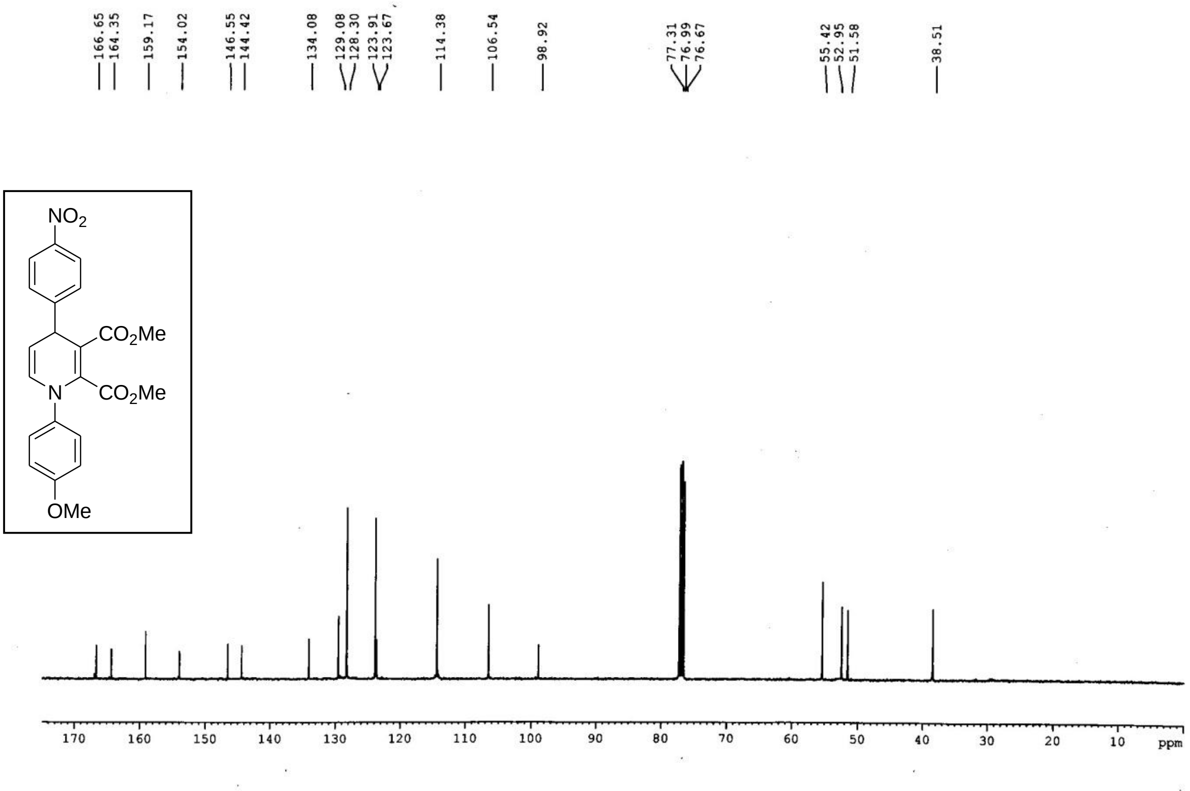

^{13}C NMR spectrum for 4j

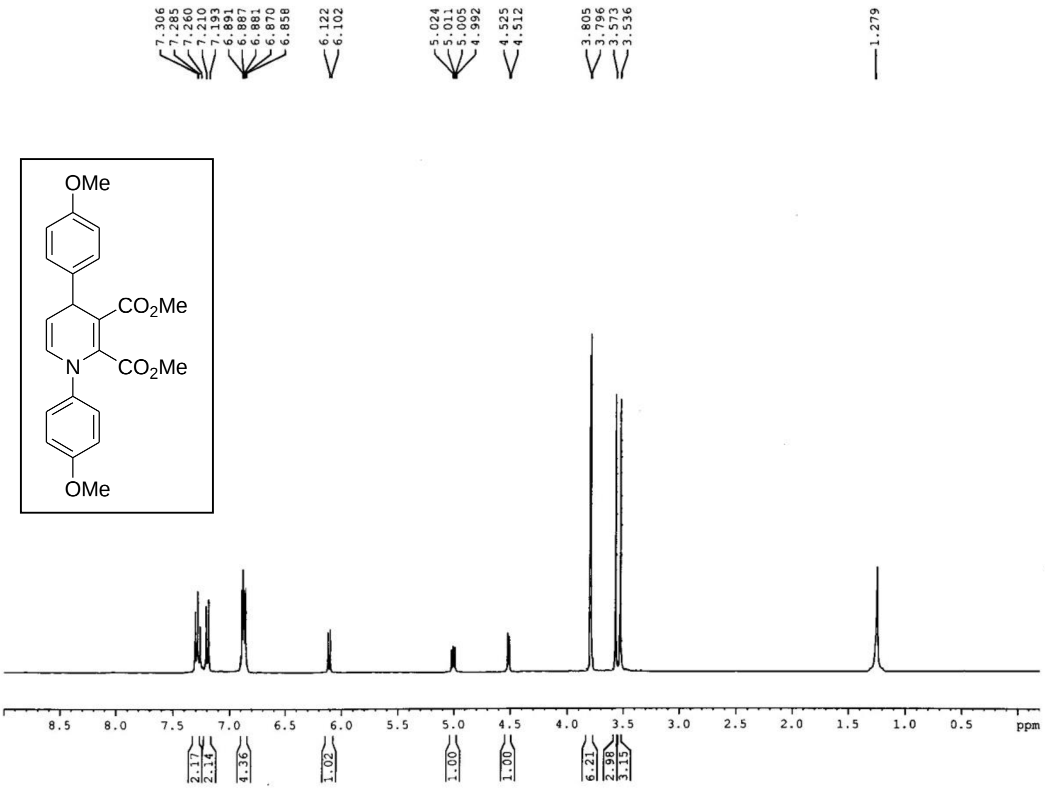

^{1}H NMR spectrum for 4k

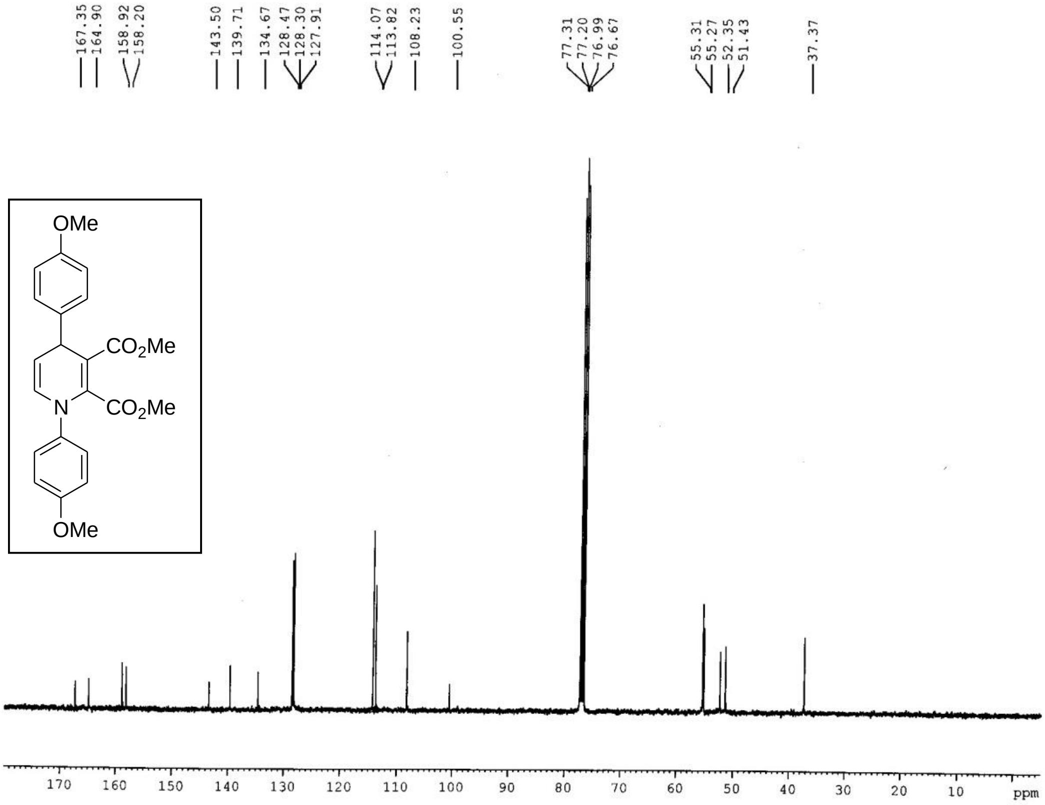

^{13}C NMR spectrum for 4k

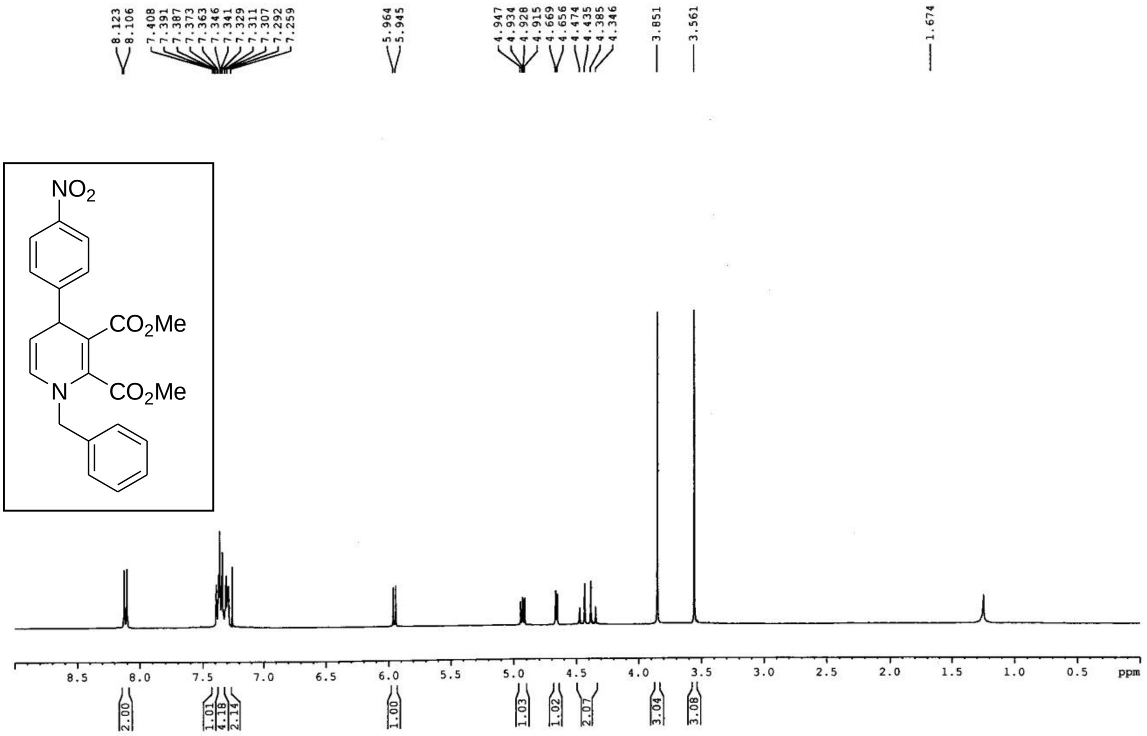

^{1}H NMR spectrum for 4l

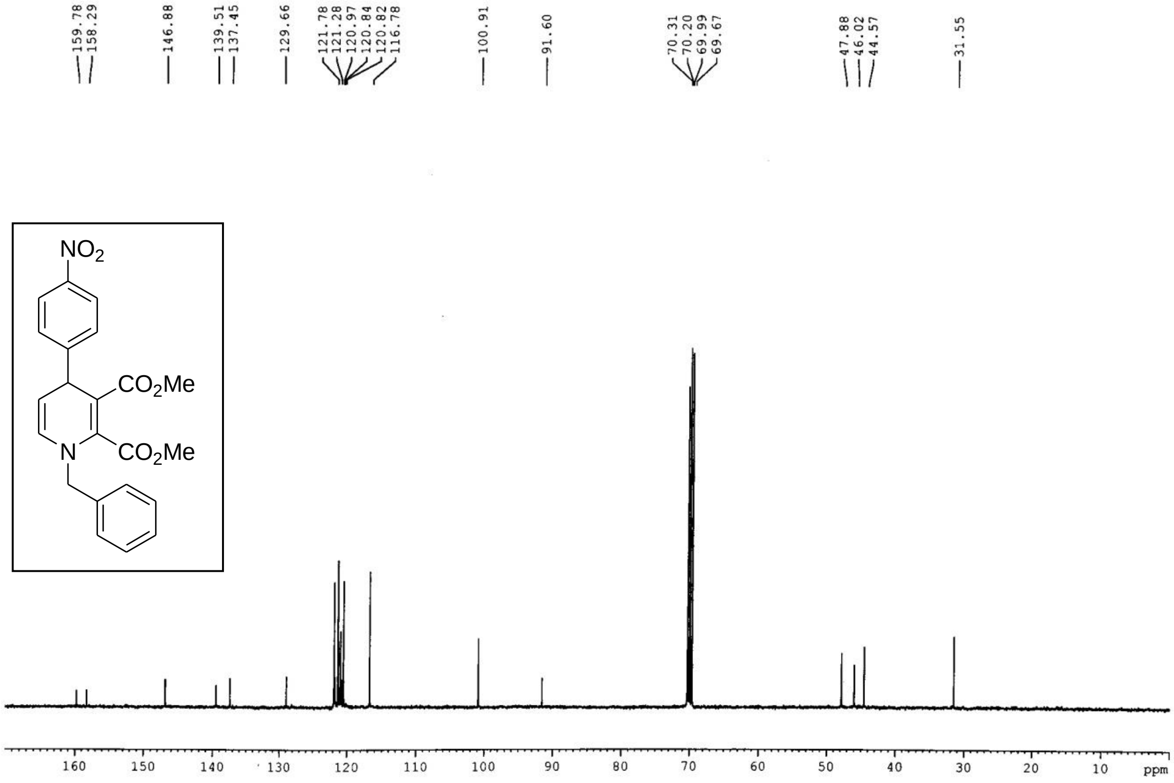

^{13}C NMR spectrum for 4l

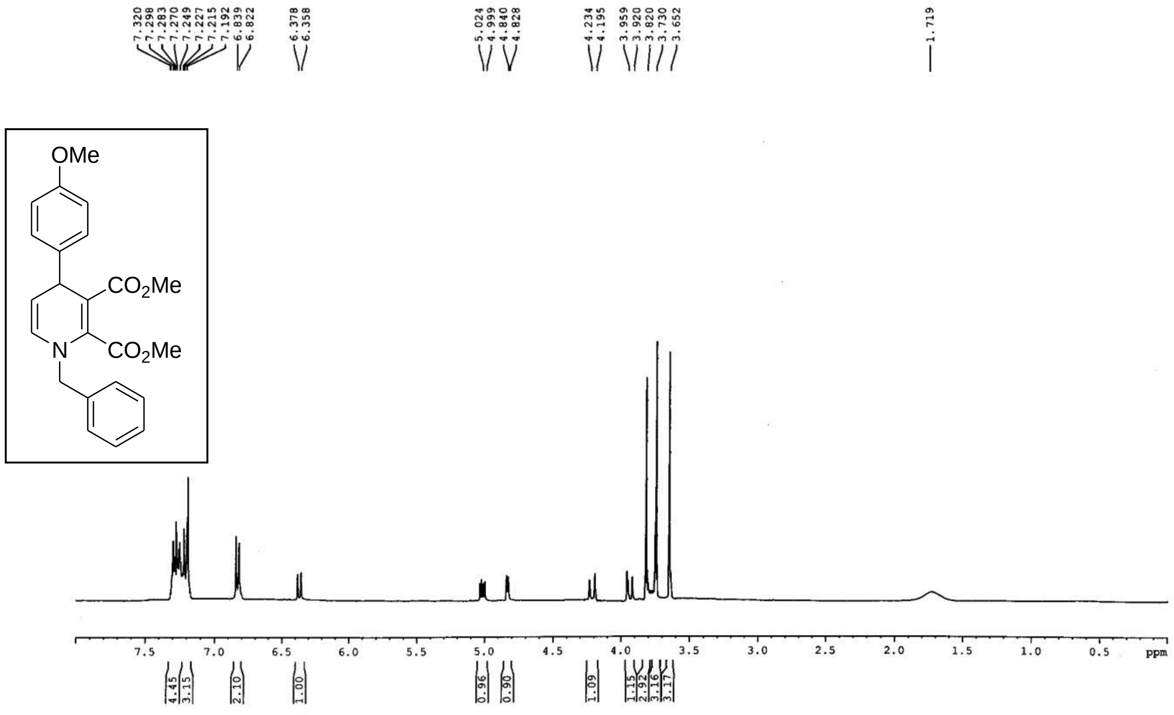

^{1}H NMR spectrum for 4m

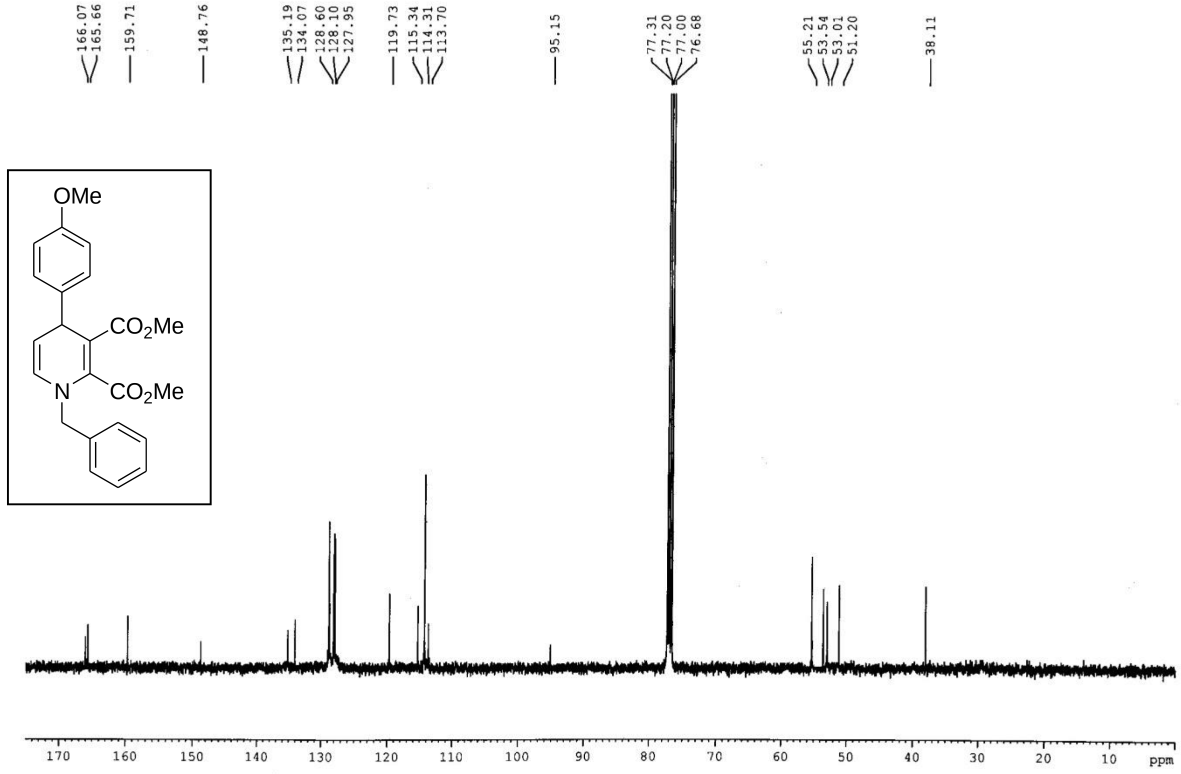

^{13}C NMR spectrum for 4m

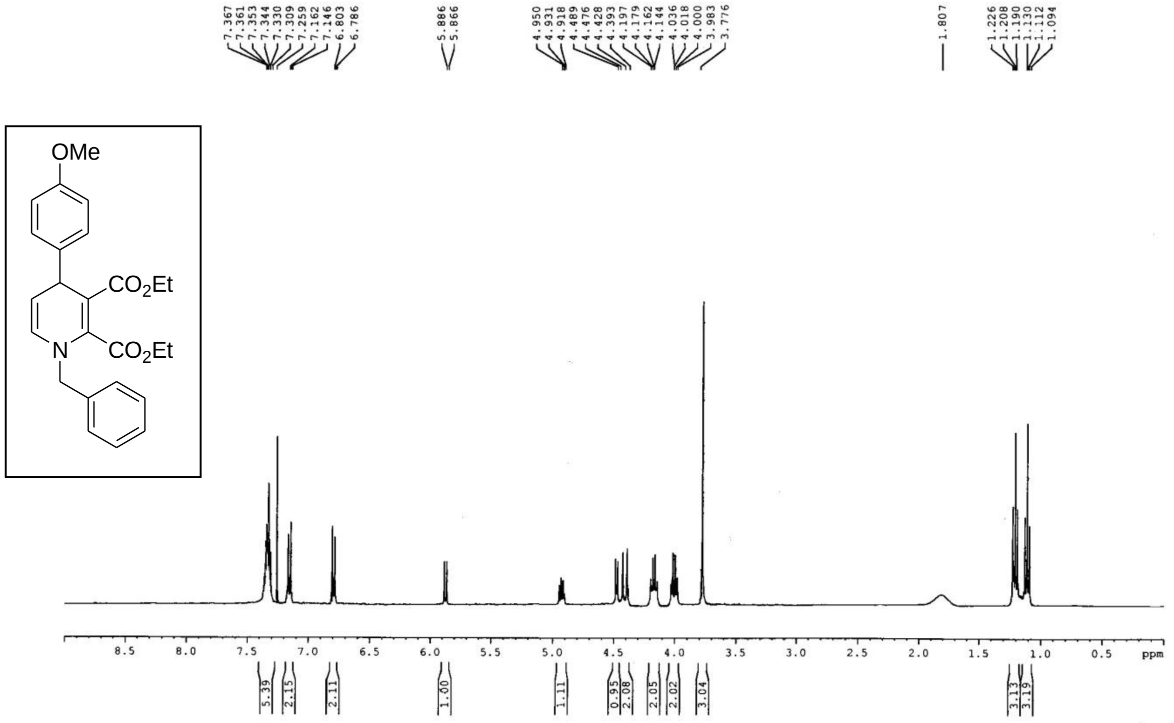

^{1}H NMR spectrum for 4n

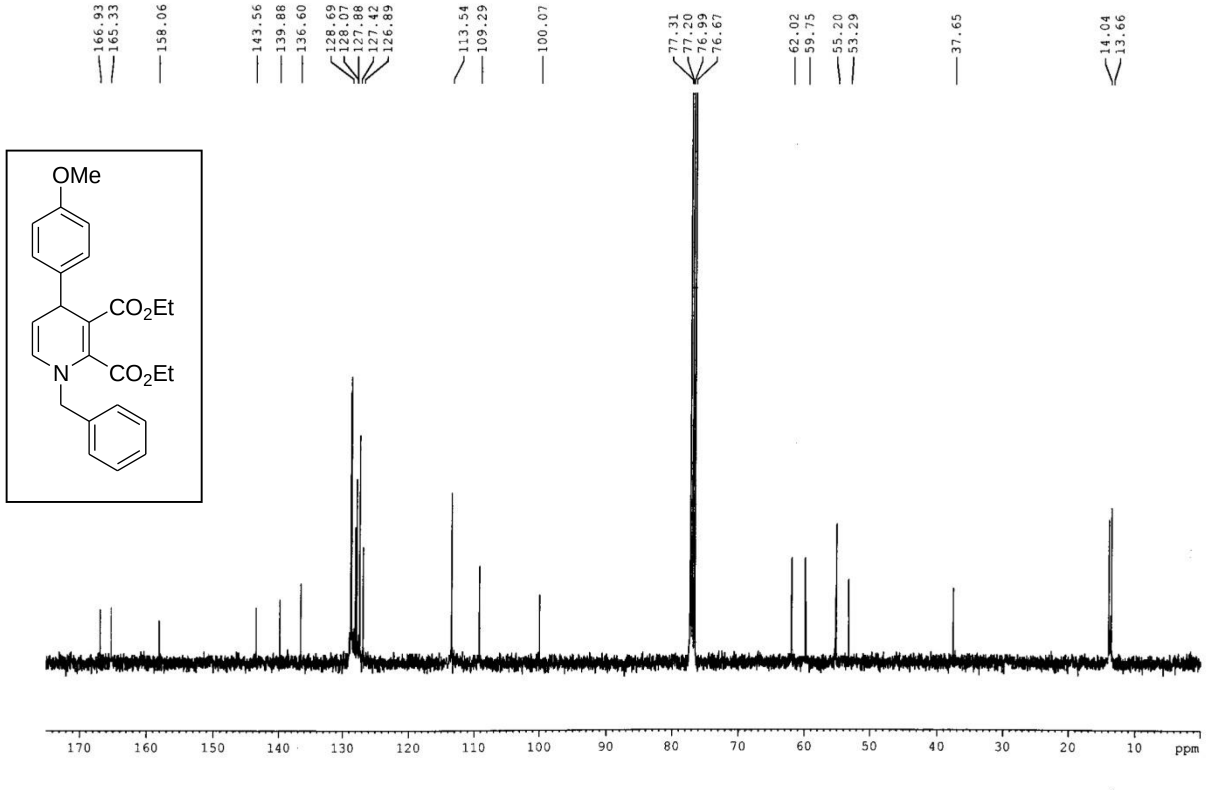

^{13}C NMR spectrum for 4n

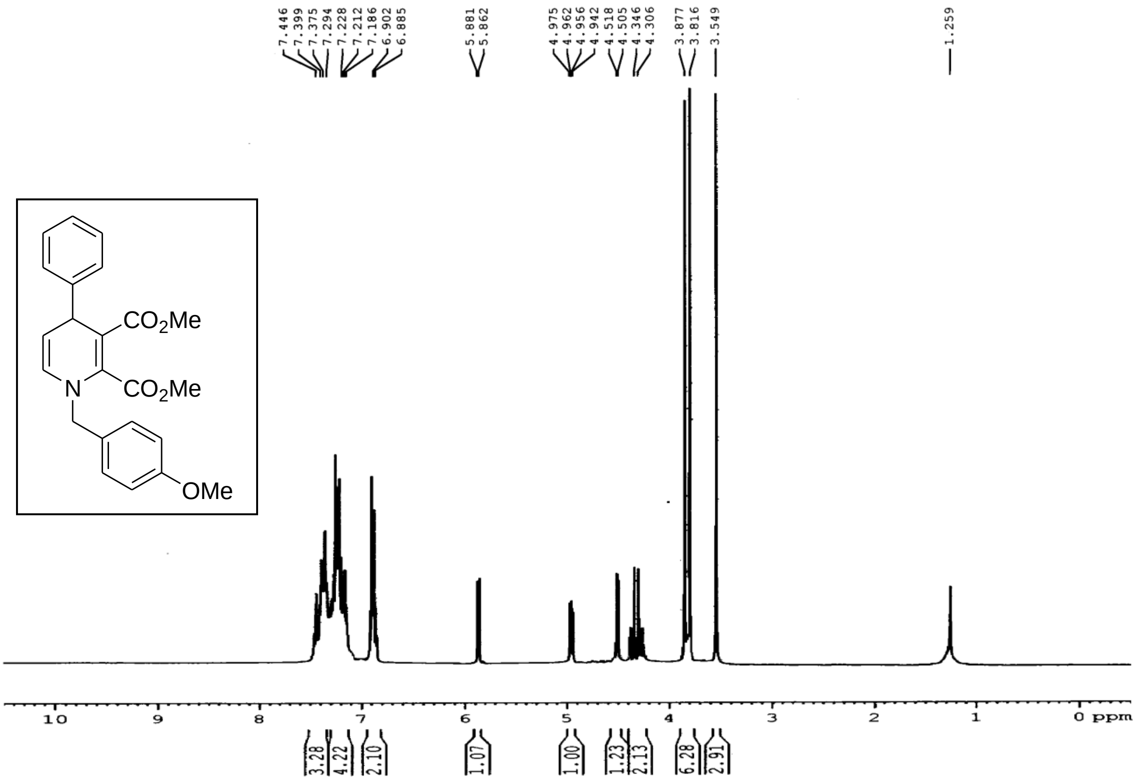

^{1}H NMR spectrum for 4o

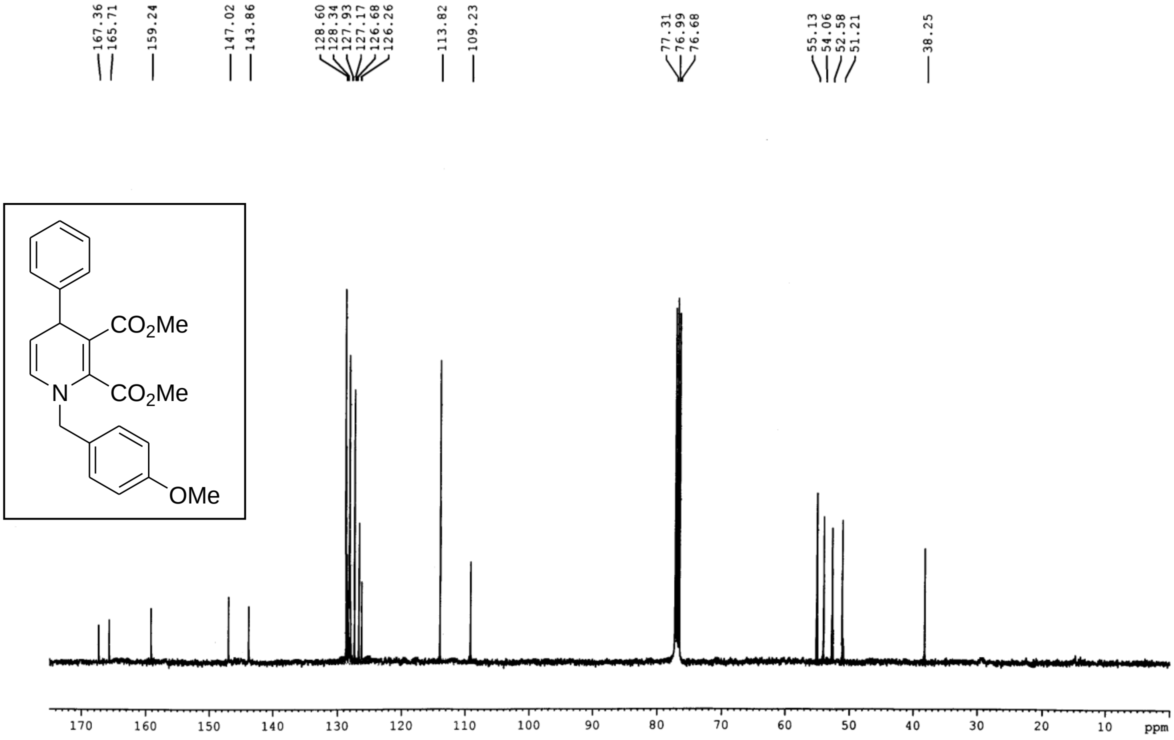

^{13}C NMR spectrum for 4o

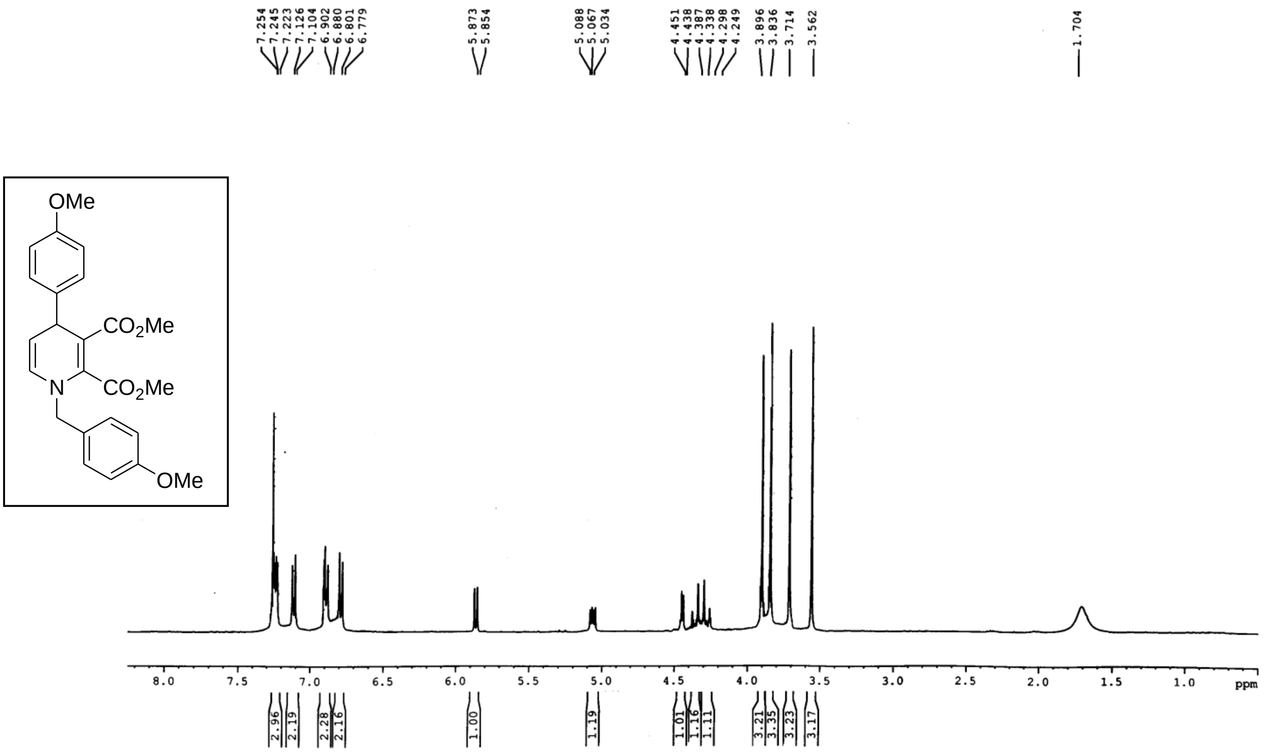

^{1}H NMR spectrum for 4p

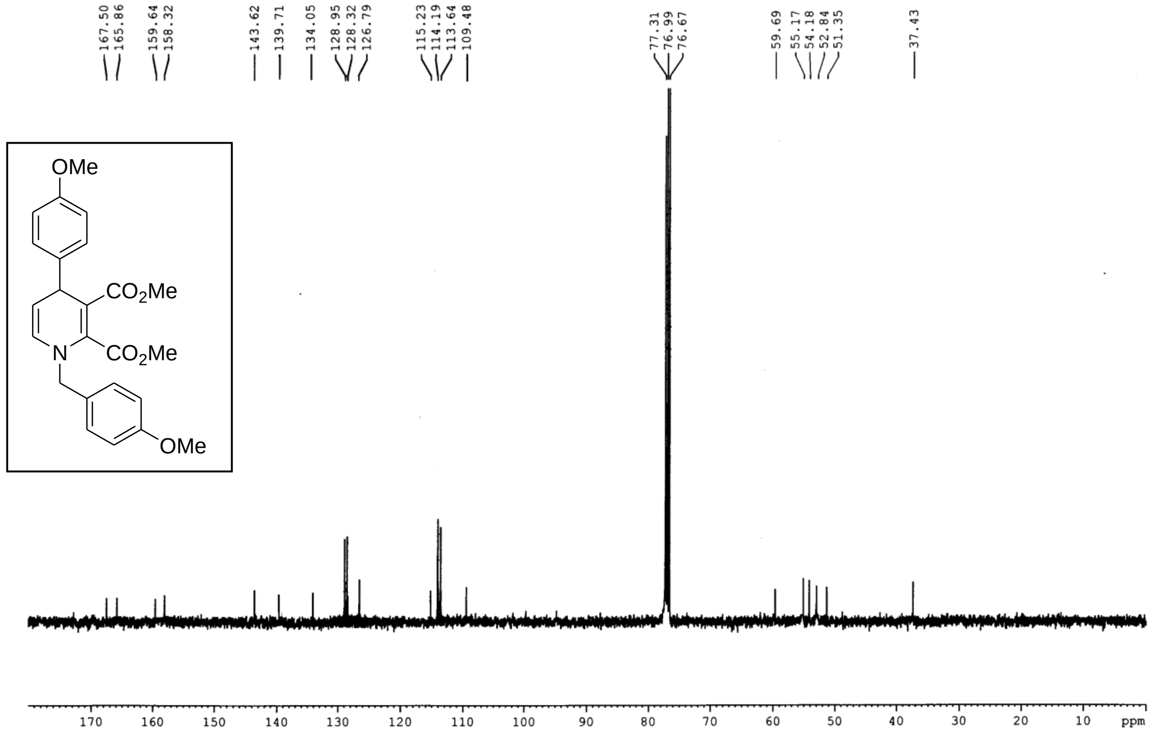

^{13}C NMR spectrum for 4p

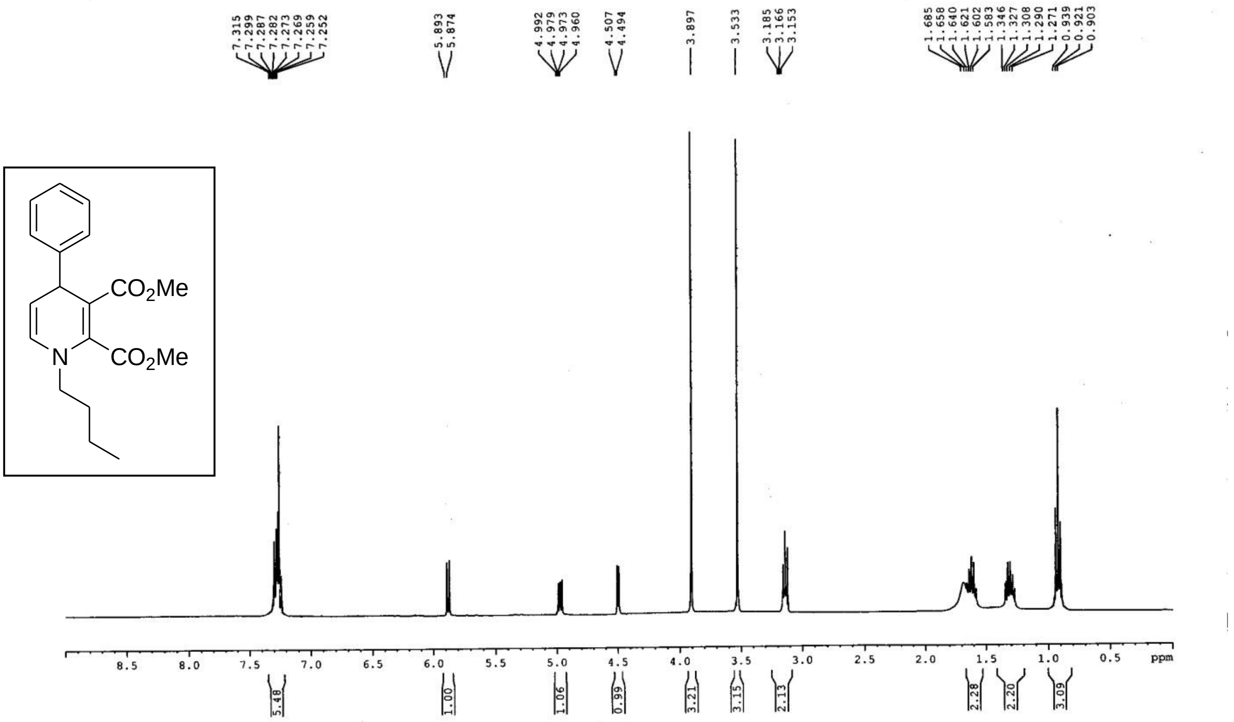

^{1}H NMR spectrum for 4q

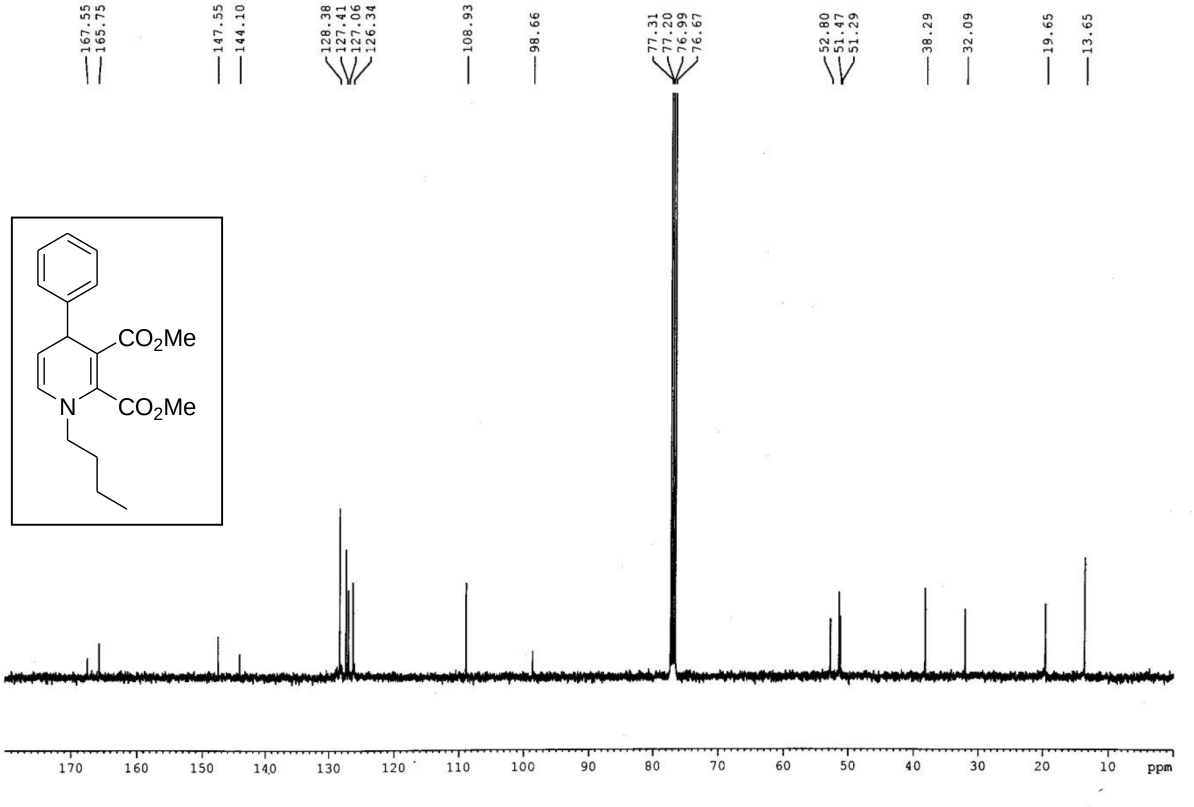

^{13}C NMR spectrum for 4q

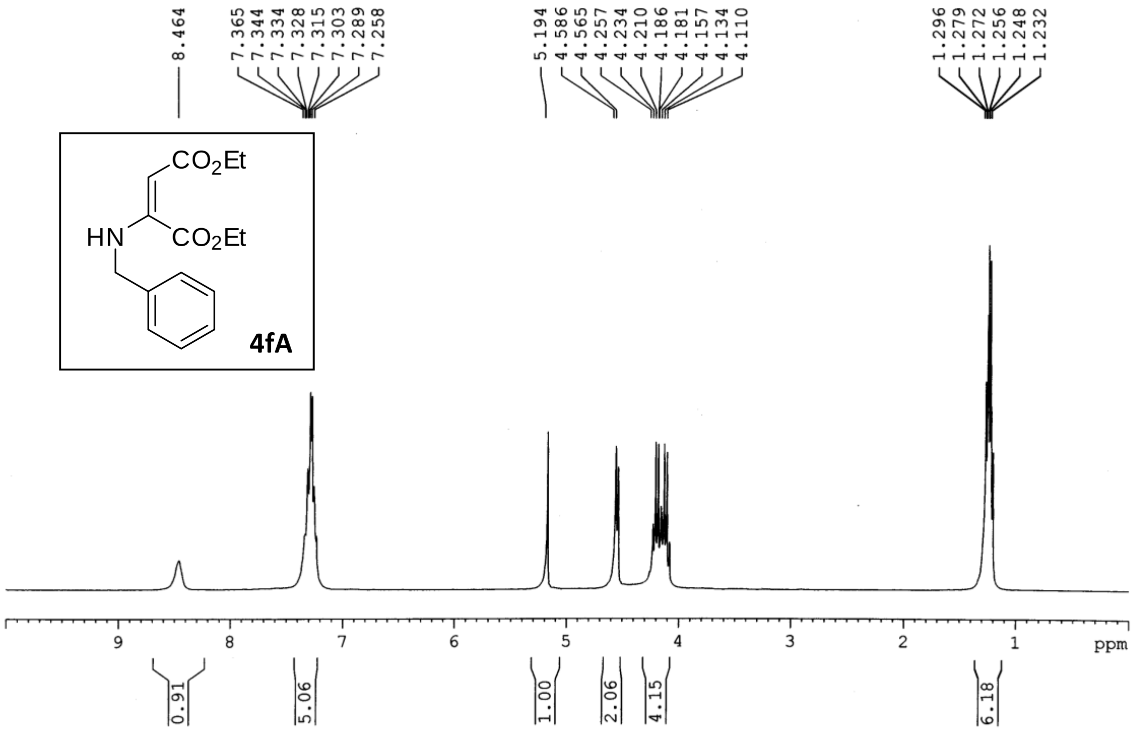

^{1}H NMR spectrum for 4fA

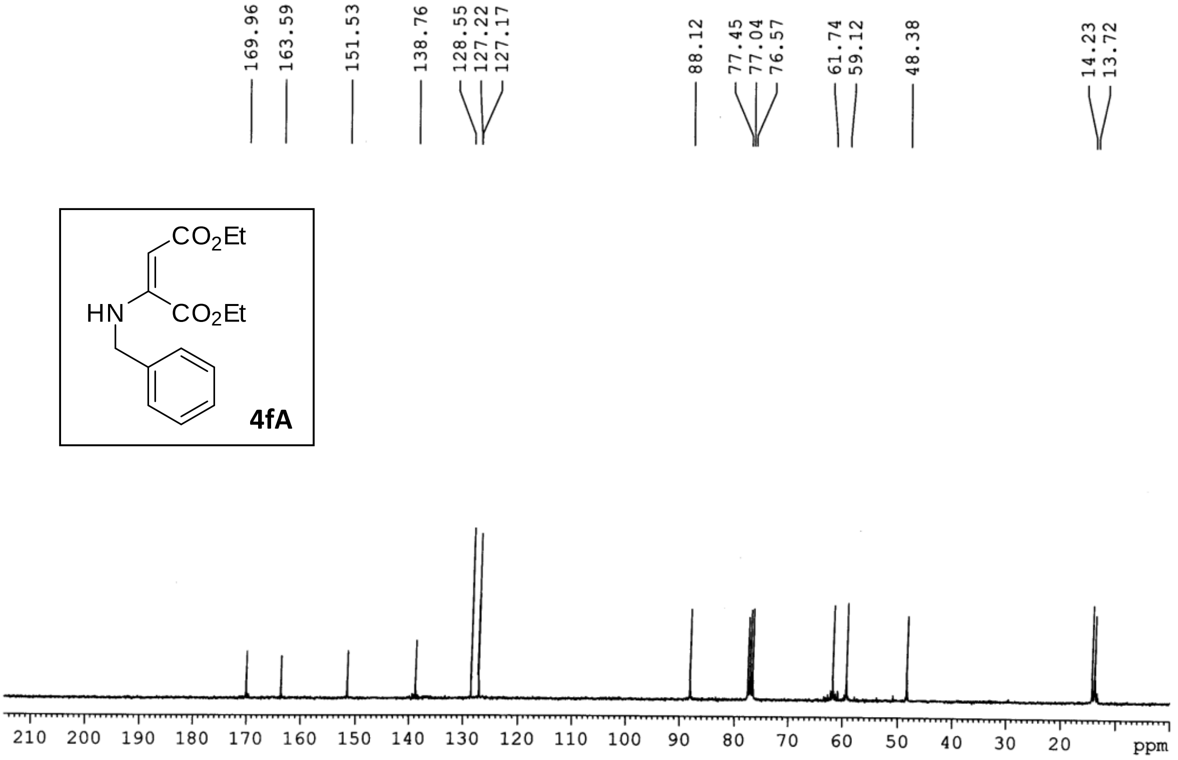

^{13}C NMR spectrum for 4fA

Crude mass spectra of compound 4k and 4m:

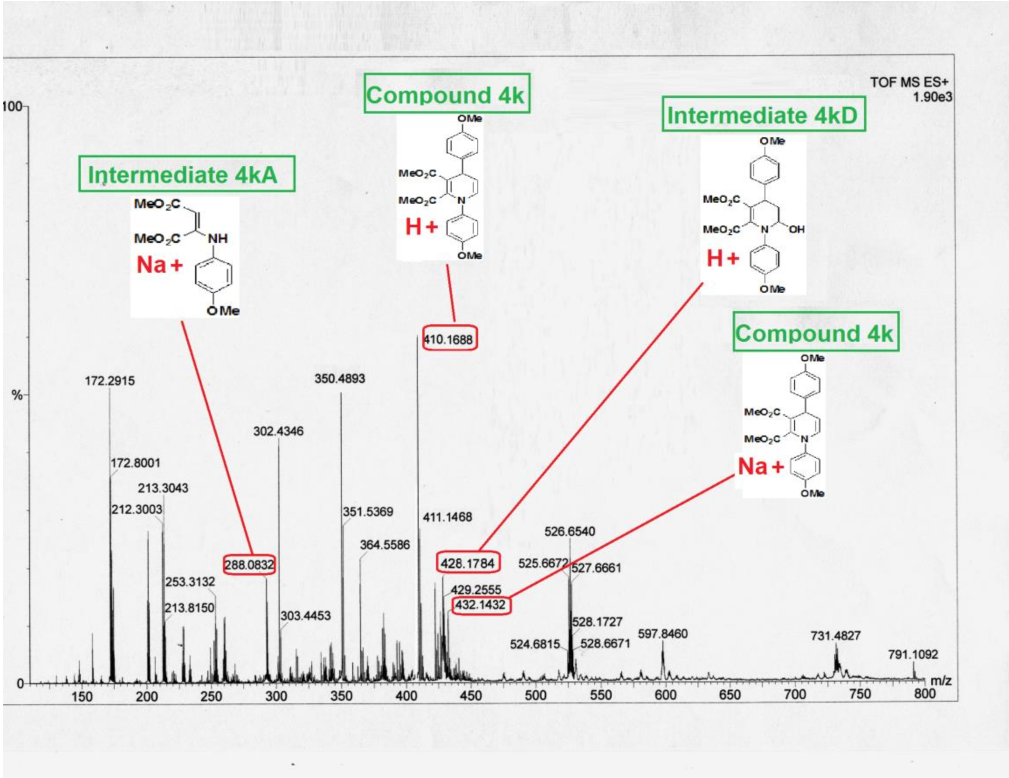

Crude mass spectrum of compound 4k

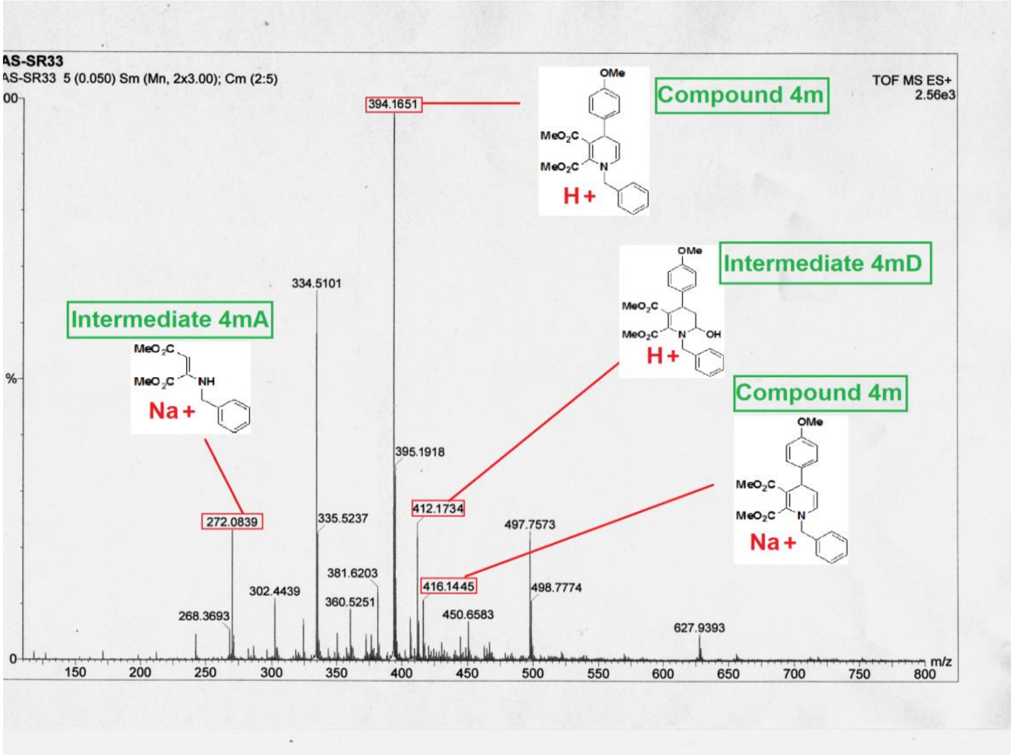

Crude mass spectrum of compound 4m
